# The molecular basis of the synergistic toxicity of nickel and copper, common environmental co-contaminants

**DOI:** 10.1128/aem.01627-25

**Published:** 2025-11-24

**Authors:** Linda Darwiche, Carlos A. Rodriguez-Bornot, Rebecca A. Ingrassia, Max J. Loccisano, Gray Waldschmidt, Jennifer L. Goff

**Affiliations:** 1 Department of Chemistry, SUNY College of Environmental Science and Forestry 14797, Syracuse, New York, USA; 2 Department of Environmental Biology, SUNY College of Environmental Science and Forestry 14797, Syracuse, New York, USA; 3 Division of Environmental Science, SUNY College of Environmental Science and Forestry 14797, Syracuse, New York, USA; 4 Department of Sustainable Resources Management, SUNY College of Environmental Science and Forestry 14797, Syracuse, New York, USA; Georgia Institute of Technology, Atlanta, Georgia, USA

**Keywords:** metabolomics, transcriptomics, stress, metals

## Abstract

Heavy metals are ubiquitous in the environment due to both natural processes and anthropogenic activities. Nickel (Ni) and copper (Cu) commonly co-occur in contaminated environments, yet most toxicity studies focus on individual metals. We investigated the combined toxicity of Ni and Cu in *Escherichia coli* using environmentally relevant concentrations of each. While each metal alone caused minimal growth inhibition, their combination was synergistically toxic. Transcriptomic and metabolomic analyses revealed unique alterations in gene expression and metabolite abundances during the combined metal treatment. Key pathways uniquely impacted by the combined metal exposure included sulfur assimilation, cysteine biosynthesis, and the tricarboxylic acid cycle. Many of these responses appeared to be linked to dysregulation of iron-sulfur (Fe-S) cluster metabolism. We observed increased expression of the genes encoding ISC Fe-S cluster assembly machinery only during metal co-treatment. Growth experiments with deletion mutants confirmed that the ISC machinery was required for survival only under the combined metal stress. We also observed the activation of a sulfur starvation response during the combined metal stress that was consistent with increased sulfur demand for Fe-S cluster biosynthesis. Deletion of *cysK*, encoding cysteine synthase, impaired growth only under combined metal exposure. Because Fe-S clusters are universal across microbial taxa, the common co-occurrence of Ni and Cu in the environment represents a widespread and underrecognized threat to microbial life and the ecosystem processes they sustain. Our findings highlight the need to further assess the effects of metal mixtures, which can trigger emergent stress responses not yet predictable from single-metal exposures.

**IMPORTANCE:**

Many environments are contaminated by metals. These metals are toxic to the microorganisms that inhabit these environments and carry out important ecosystem services. While much is known about bacterial responses to single metal stress, in most contaminated environments, metals typically exist as mixtures. Nickel (Ni) and copper (Cu) are common co-contaminants. We tested Ni and Cu in combination to examine the mechanism behind their synergistic toxicity in the model bacterium *Escherichia coli* K-12. We found that the two metals in combination are likely disrupting iron-sulfur (Fe-S) clusters. Since Fe-S clusters are ubiquitous across microbial taxa and critical for microbial metabolism, this suggests that these two common co-contaminants may be toxic to diverse microorganisms.

## INTRODUCTION

Globally, heavy metal contamination has increased significantly since the Industrial Revolution began in the 19th century (1). Although heavy metals are needed in trace amounts for biological processes such as electron transport, metabolism, and enzyme regulation (2), at higher concentrations, all heavy metals are toxic (3). High levels of heavy metals can be harmful to living organisms, including bacteria performing key ecosystem services (4–10).

The presence of heavy metal ions in the environment impacts bacterial biochemistry. Mechanisms of metal ion toxicity in bacteria include metalloenzyme mismetallation (i.e., insertion of the incorrect metal cofactor) (11), antagonism of essential metal ion uptake (12), generation of reactive oxygen species (ROS) (13), and induction of disulfide stress (14). Bacteria have numerous resistance mechanisms that allow them to survive this heavy metal stress. These mechanisms are intrinsic or acquired by chromosomal mutations or through transferable genetic materials (15–19) and include increased expression of efflux pumps, decreased expression of uptake systems, increased expression of metal-binding proteins, and increased expression of metal-reducing oxidoreductases—among others (15). This body of literature describing mechanisms of heavy metal toxicity and resistance in bacteria is largely derived from studies conducted with individual metal exposures. However, in most environments, heavy metal contamination typically does not involve just one individual metal. Rather, heavy metals tend to exist as contaminant mixtures (20–22).

Several studies have highlighted the synergistic and antagonistic effects of metal combinations on bacterial growth (23–29) but did not examine the underlying molecular mechanisms of these effects. In a recent study, we utilized an environmental *Bacillus cereus* isolate—originating from a multi-metal-contaminated site—to examine the effects of a site-informed eight-metal mixture on cell physiology through a combination of proteomic and metabolomic analyses (30). We observed that the metal mixture elicited a unique physiological response that could not be predicted by the single metal exposures alone. In analyzing the proteome shifts across all stress conditions (metal mixture exposure and eight individual metal exposures), we found that 65% of differentially abundant proteins were differentially abundant only under the metal mixture stress. These findings highlighted the limitations of single metal stress experiments in fully understanding the mechanisms by which bacteria respond to heavy metal stress in their environment. However, disentangling metal interactions in a non-model bacterial system is challenging—further compounded by the difficulty in studying an eight-metal mixture. Thus, there is a need to develop a foundational, predictive understanding of metal interactions using simplified combinations of heavy metals in model organisms. We chose *Escherichia coli* K-12 for this work as it is a well-characterized system for studying metal homeostasis in bacteria (31, 32).

Nickel (Ni) and copper (Cu) are two commonly co-occurring heavy metals in the environment (33, 34), often introduced through anthropogenic activities (35). While each metal, individually, has distinct toxicological effects, their interactions within bacterial systems remain underexplored. An early study described the synergistic toxicity of Ni and Cu in *Bacillus subtilis, Enterobacter aerogenes,* and *Nocardia corallina* (33). However, no mechanistic details were provided, and there was no follow-up on this work. Additionally, our recent study with the *B. cereus* isolate suggested that the synergism between Ni and Cu was a significant contributor to the overall toxicity of the eight-metal mixture (30). In this current study, we utilized environmentally relevant concentrations of Ni and Cu to confirm their synergistic toxicity in *Escherichia coli* K-12, our model system for this analysis. Utilizing a combination of transcriptomics, metabolomics, and screens of mutant strains, we examined how exposure to a combination of Ni and Cu impacts *E. coli* growth and functioning, providing a deeper understanding of their combined impact on microbial physiology.

## MATERIALS AND METHODS

### Bacterial strains

*Escherichia coli* K-12 BW25113 was used throughout the study, unless otherwise indicated. Strain BW25113 is the parental strain for the *E. coli* Keio Knockout Collection (36). Both the parental and mutant strains were purchased from Horizon (Cambridge, UK).

### Media preparation

To prepare the LB liquid medium, 10 g of tryptone (IBI Scientific, USA), 10 g of NaCl (VWR, USA), and 5 g of yeast extract (MP Biomedicals, USA) were dissolved in 950 mL of deionized water (DI H_2_O). The solution was mixed until the solutes were fully dissolved, then the final volume was adjusted to 1 L by adding more distilled water. The medium was sterilized by autoclaving. For preparing LB agar plates, 15 g of agar was added to the solution before autoclaving to allow the medium to solidify. MES Buffered Minimal Medium (MBMM) was modified from Rathnayake et al. (37). The full recipe can be found under Supplemental methods section in the supporting information document.

### Heavy metals stock solutions

Nickel (II) (Ni) chloride hexahydrate (MP Biomedicals, USA) was used to prepare 50 mM stock solution using DI H_2_O. Copper (II) (Cu) chloride dihydrate (Fisher Scientific, USA) was used to prepare a 50 mM stock solution using DI H_2_O.

### Bacteria and growth conditions

For experimental work, glycerol stocks of *Escherichia coli* K-12 BW25113 (the parent strain of the Keio collection) and Keio collection mutants (36) were streaked onto LB plates and grown overnight at 37°C. A full list of the strains used can be found in Table S1. Two to three colonies were selected and transferred to a liquid medium and then incubated in a shaker incubator at 37°C for 24 hours under constant shaking to allow bacterial growth before transfer into experimental growth medium.

### Bacterial growth curves

To evaluate the effects of specific supplements on *Escherichia coli* growth under metal stress, wild-type *E. coli* was cultured overnight in LB medium at 37°C with shaking at 200  rpm. The next day, cultures were diluted 1:200 into MBMM and subjected to treatment with 30  µM NiCl₂, 15  µM CuCl₂, or a combination of both metals. Supplement additions, if utilized, were performed immediately after metal treatment. The experimental design included a control (no metal), one of three metal treatments (30 µM Ni, 15 µM Cu, or 30 µM Ni and 15 µM Cu), and each metal treatment in combination with the supplement additions (if used).

A range of concentrations was tested for each supplement to identify the most effective dose in restoring growth under metal stress conditions. These concentrations were selected based on their concentrations in common bacterial growth media, such as LB. For cystine, 10 concentrations (0.2, 0.4, 0.6, 0.8, 1.0, 1.2, 1.4, 1.6, and 1.8 mM) were tested, and 0.8 mM was selected as the working concentration. For sulfate, five concentrations (2, 5, 10, 15, and 20 mM) were tested using magnesium sulfate heptahydrate, and 20 mM was selected as the working concentration. For glutathione, four concentrations (1, 2, 3, and 5 mM) were tested, and 1 mM was selected as the working concentration. For methionine, five concentrations (3, 5, 7, 10, and 12 mM) were tested, and 7 mM was chosen as the working concentration. For histidine, 10 concentrations (0.3, 0.4, 0.5, 0.6, 0.8, 1.0, 1.2, 1.4, 1.6, and 1.8 mM) were evaluated, and 0.3 mM was selected as the working concentration. Iron supplementation was delivered using iron (II) sulfate heptahydrate, chelated with citrate in a 3:2 ratio (Fe:citrate). Five concentrations of Fe²^+^ (3, 6, 12, 25, and 50 mM) were tested.

Following treatment, 200 µL of each culture was transferred into wells of a 96-well microtiter plate. The microplate was placed in a BioTek Synergy HTX plate reader, which monitored the optical density (OD) at 600 nm (OD_600_) while shaking and incubating the cultures at 37°C. Readings were taken by the plate reader every 30 minutes for 24 hours.

### Sample preparation for transcriptomics and metabolomics

For transcriptomic and metabolomic analyses, bacterial cultures (triplicates for transcriptomics and quintuplicates for metabolomics) were prepared as described above, with a 1:200 dilution in MBMM and grown until they reached an OD of 0.25–0.3 (mid-exponential phase). Cultures were treated for 2 hours with the following conditions: (i) Control (no treatment), (ii) 30 µM Ni, (iii) 15 µM Cu, and (iv) 30 µM Ni and 15 µM Cu. After treatment, cultures were centrifuged at 10,000 rpm for 5 minutes, the supernatant was discarded, and the cell pellets were washed twice with phosphate-buffered saline (PBS). The washed pellets were resuspended in 1 mL of PBS, transferred to 2 mL sterile Eppendorf tubes, and centrifuged again. For metabolomic analyses, the final pellet was frozen at −80°C until shipment for analysis. For transcriptomics, RNA was immediately extracted using the Zymo Quick-RNA Miniprep Kit, following the manufacturer’s protocols. RNA samples were immediately frozen at −80°C prior to further analysis.

### Library preparation and RNA sequencing

Library preparation and RNA sequencing were performed at Biomarker Technologies (BMKGENE) USA Inc. (Durham, NC, USA) as described in the Supplemental methods section in the supporting information document.

### Transcriptome assembly and analysis

The RNA-seq analysis was performed using the US Department of Energy’s KnowledgeBase (KBase) (38) bioinformatics platform. A standardized workflow incorporating HISAT2 (39), StringTie (40), and DESeq2 (41) was followed. First, the *Escherichia coli* K-12 reference genome (RefSeq: NC_000913) was imported for alignment. Next, raw sequencing reads were obtained from an online repository and organized into a structured RNA-seq sample set. Read quality was assessed using FastQC (42) to identify potential sequencing artifacts. The reads were then aligned to the reference genome using HISAT2. Following alignment, transcripts were assembled using StringTie to reconstruct gene structures. Differential expression analysis was performed using DESeq2 to identify genes with significant expression changes. Finally, a filtered differential expression matrix was generated to refine the data set for downstream functional analysis. The final differential expression data set can be seen in Table S2. Transcripts per million (TPM) counts for each replicate can be seen in Table S3. Finally, raw read data are deposited in the Sequence Read Archive under the accession numbers SRR34735813–SRR34735824.

### LC-MS metabolomics

The liquid chromatography-mass spectrometry (LC-MS)-based untargeted metabolomic analysis was performed at Creative Proteomics (Shirley, NY, USA). Extraction and analysis methods are described in the Supplemental methods section in the supporting information document.

### Metabolomic data analysis

For the metabolomics analysis, *P*-values were initially calculated using a Welch’s t-test to compare metabolite concentration averages between each of the metal treatments and the control. This was followed by adjustment for multiple comparisons using the Benjamini-Hochberg procedure.

### ROS assay

2′,7′-Dichlorodihydrofluorescein diacetate (H2DCFDA) is a non-fluorescent molecule. Once imported into the cell, it is deacetylated to dichlorofluorescein (DCFH). When DCFH is exposed to ROS, the molecule is oxidized to the fluorescent dichlorofluorescein (DCF) (43). To prepare the probe, 5 mg of H2DCFDA was dissolved in 5 mL DMSO to prepare a 2 mM solution, which was stored at −20°C. To measure ROS production, an overnight culture of the wild-type *E. coli* K-12 was diluted 200-fold into MBMM and left to regrow at 37°C in a shaking incubator until mid-log growth phase (OD_600_ of 0.25–0.3). Mid-log cultures were centrifuged at 5,900 × *g* for 5 minutes. The pellet was resuspended in sterile MBMM. The H2DCFDA probe was added at a final concentration of 5 µM. The cells were incubated with the probe for 1 hour at room temperature in the absence of light to allow uptake of the probe into bacterial cells. After incubation, the culture was centrifuged and resuspended in sterile MBMM. The cells with the probe were then exposed to their respective treatments (30 mM Ni, 15 mM Cu, and the mixture of the two) and monitored for fluorescence signal in our incubating plate reader. A positive control was performed by treating the H2DCFDA-treated cells with a final concentration of 3% H₂O₂. The fluorescence signal was monitored for 12 hours with excitation and emission wavelengths of 485/20 and 528/20 nm, respectively, using the bottom light source. Measurements were recorded every 15 minutes.

### Thiol quantification by Ellman’s assay

Reduced thiol concentrations were quantified using Ellman’s reagent (5,5′-dithiobis(2-nitrobenzoic acid), DTNB) (44). A reduced glutathione (GSH) standard curve was prepared by generating 11 serial twofold dilutions in Tris-HCl buffer (50 mM, pH 8.0). The dilution series ranged from 1 mM down to ~1.95 µM GSH and included a buffer-only blank (0 µM). For each dilution, 100 µL of 0.2 mM DTNB was added to 100 µL of GSH standard in a 96-well microplate. Reactions were incubated at 37°C for 30 min in the dark to allow complete chromophore development. Absorbance was then measured at 412 nm using a microplate reader. The standard curve (A412 vs. [GSH]) was generated by subtracting the blank absorbance and plotting mean values from replicate wells.

*E. coli* cultures were inoculated into 3 mL of MBMM medium and incubated at 37°C with shaking at 200 rpm until reaching OD_600_ = 0.25–0.3. At this point, cultures were treated for 2 hours with the following conditions: (i) Control (no treatment), (ii) 30 µM Ni, (iii) 15 µM Cu, and (iv) 30 µM Ni and 15 µM Cu. Cells were then harvested by centrifugation at 10,000 rpm for 5 min, and supernatants were discarded. Pellets were washed once with PBS before being resuspended in 1 mL of extraction buffer (50 mM Tris-HCl, pH 8.0, 5 mM EDTA, 0.1% SDS, 0.1 mM DTNB). Cell suspensions were vortexed briefly and incubated at 37°C for 30 min in the dark. After incubation, samples were centrifuged at 13,000 g for 2–3 min to remove debris. The clarified supernatant was transferred to 96-well plates, and absorbance was measured at 412 nm. Thiol content in cell extracts was determined by comparing with the GSH calibration curve. Absorbance values from samples were first blank-corrected (0 µM GSH) before interpolation. Thiol concentrations were expressed as equivalents of reduced glutathione.

### Spot dilution assay

Spot-plating was performed to evaluate metal sensitivity of the wild-type *E. coli* and selected mutants from the Keio collection (Table S1). Overnight cultures were grown in LB medium at 37°C with shaking at 200  rpm. The following day, cultures were diluted into sterile PBS to an OD_600_ of 0.45. Tenfold serial dilutions (10⁰–10⁻⁵) were prepared in PBS, and 5 µL of each dilution was spotted in duplicate onto MBMM agar supplemented with the following: (i) control (MBMM without metal supplementation) (ii), 30  µM NiCl₂, (iii) 15  µM CuCl₂, and (iv) combined treatment with 30 µM NiCl₂ and 15 µM CuCl₂. Plates for the control and single-metal treatments were incubated at 37°C for 24 hours, while plates containing the combined metal treatment were incubated at 37°C for 6 days to allow for the colonies to grow to a sufficient size to photograph—even though growth phenotypes were visible much earlier than 6 days. Plates were photographed using a smartphone camera under consistent lighting conditions. Experiments with the mutant strains and the wild-type strain (as a control) were performed in parallel at least two times to verify phenotypes, with one representative experiment shown.

## RESULTS AND DISCUSSION

### Ni and Cu commonly co-occur in freshwater environments

**Fig 1:**
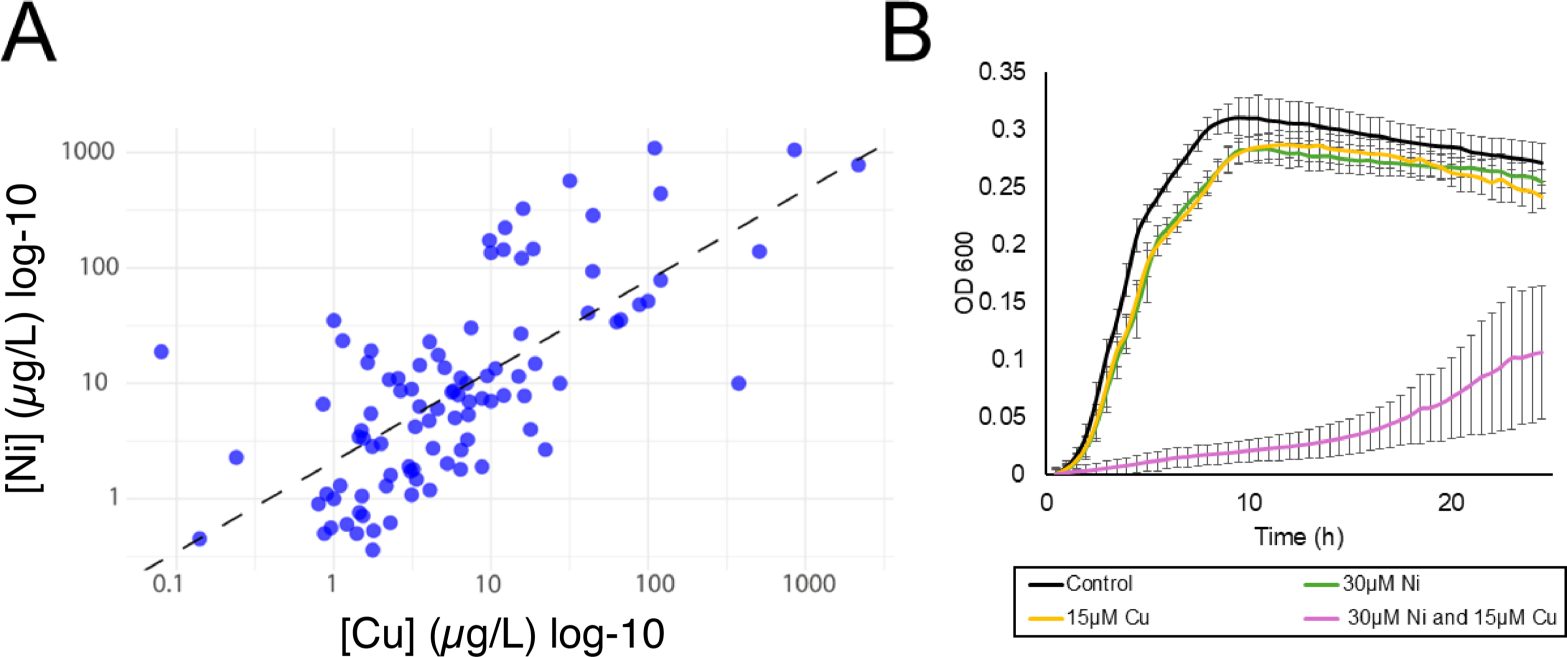
(**A**) Log–log correlation between Cu and Ni concentrations (µg/L) in global river and lake water bodies based on compiled environmental monitoring data from 1972 to 2017 (21). The regression line for the equation [Ni] =2.107[Cu]^0.784^ (R^2^ = 0.493, *P* = 1e-15) is shown as a dashed line. One µg/L Ni equals 0.017 µM Ni, and 1 µg/L Cu equals 0.016 µM Cu. (**B**) Growth curves of *E. coli* under control conditions and treatment with 15 µM Cu, 30 µM Ni, and combined Ni and Cu exposure. Each point represents the average of three replicates, and error bars represent ±SD.

We investigated the individual and combined effects of Ni and Cu on *E. coli* as these two metals are commonly detected together in polluted ecosystems, particularly in areas influenced by industrial discharge, mining activities, and agricultural runoff (45, 46). We first reanalyzed a data set of heavy metal concentrations in 239 global rivers and lakes from 1972 to 2017 (21). We performed a log-log linear regression to evaluate the power-law relationship between [Ni] and [Cu] in this data set. Our analysis revealed a statistically significant, positive correlation between the concentrations of Cu and Ni (Fig. 1A).

### Synergistic toxicity of Ni and Cu

We next examined the growth of *E. coli* with a range of environmentally relevant concentrations of Ni^2+^ and Cu^2+^ to define our treatment conditions. Ni concentrations in freshwater have been reported between 0.006 and 157 µM (0.352–9,216 µg/L) (21, 47, 48), and Cu concentrations in freshwater range from 0.0126 to 432 µM (0.813–27,864 µg/L) (21, 47–50). While Cu^2+^ was used for the amendments, it is reduced to Cu^1+^ intracellularly (51). In our experimental system, we use “Ni” to refer to Ni^2+^ and “Cu” to refer to both the Cu^2+^ added to the cultures and the intracellular Cu^1+^, with distinction between the two oxidation states made as needed. Our growth medium was modified from Rathnayake et al. and utilizes minimal nutrient concentrations, such as phosphate, to maximize free metal ion availability (37). As shown in Fig. S3, the growth of *E. coli* treated with 20 µM Ni and 10 µM Cu was comparable to the untreated control with minor growth inhibition, indicating minimal toxicity. In contrast, treatment with 40 µM Ni and 20 µM Cu resulted in a significant reduction in bacterial growth. Based on these observations, an intermediate concentration of 30 µM Ni and 15 µM Cu was selected for further experiments. This combination was chosen to avoid lethality while still exerting a significant inhibitory effect on bacterial growth. We treated *E. coli* with either 30 µM Ni or 15 µM Cu, individually. These treatments resulted in minimal growth defects (Fig. 1B). However, when the two metals were added in combination at the same concentrations (30 µM Ni and 15 µM Cu), a significant decrease in *E. coli* growth occurred (Fig. 1B), suggesting that the toxicity of Ni and Cu in combination is synergistic, as described in previous studies (33, 52).

To characterize the cellular response to the combined Ni and Cu treatment, we conducted transcriptomic and metabolomic analyses of *E. coli* cultures treated with Ni (30 µM), Cu (15 µM), or their combination (30 µM Ni and 15 µM Cu). Untreated cultures served as controls. Changes in gene expression for each metal treatment were determined relative to the control after 2 hours of exposure during mid-log growth. This timing ensured that all cultures were more synchronized in the growth phase than what we observed in Fig. 1B and had sufficient biomass for RNA extraction. As a result, the kinetics of this experiment differ somewhat from those shown in Fig. 1B. In these cultures, cells exposed to the metal mixture stopped increasing in OD_600_ immediately after exposure. The Cu-treated and control cultures continued to grow at similar rates, although Cu exposure caused a mild reduction in final biomass (Fig. S1). In contrast, Ni-exposed cultures exhibited an initial lag in growth following the exposure relative to the control but subsequently grew more rapidly, ultimately reaching a biomass comparable to that of the Cu-treated cultures. This lag in the Ni-exposed cells was a phenotype that we did observe on occasion during our other growth experiments, an example of which can be seen in Fig. S2. We suspect that these differences reflect minor batch-to-batch variation in the growth medium (53) or small differences in the physiological state of the inoculum at the time of exposure (54).

**Fig 2:**
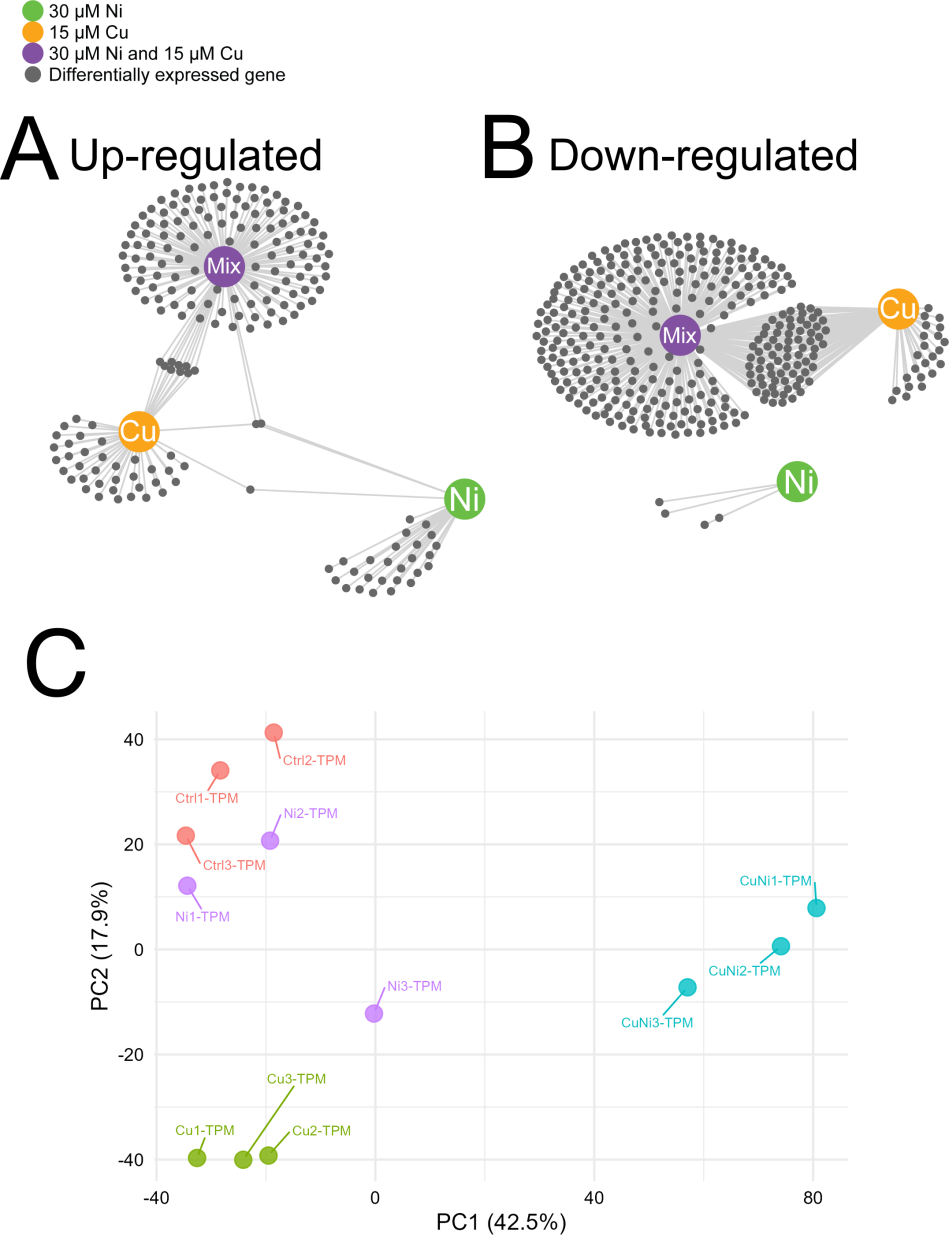
Transcriptomic analysis of *E. coli* reveals gene expression changes induced by 15 µm Cu, 30 µm Ni, and their combination. The network diagram shows differentially expressed (small, gray nodes) genes under each metal treatment (large, colored nodes). Edges connect treatment nodes to gene nodes. (Panel **A**: upregulated genes; Panel **B**: downregulated genes). For each condition, three replicates were performed. (**C**) PCA plot of transcriptomic data. Each sample’s reads in TPM are compared against each other. The distance between points represents dissimilarity.

In total, there were 512 differentially expressed genes across the three metal treatments (Table S2), of which 360 (70% of total) were uniquely differentially expressed in the combined metal treatment (Fig. 2). Principal component analysis (PCA) revealed that the transcriptome from the combined metal exposure was clearly separated from all other treatments along PC1 (accounting for 42.5% of variance) (Fig. 2C). The control, Ni-exposed, and Cu-exposed transcriptomes were further separated along PC2 (17.9% of variance). Untargeted metabolomic profiling revealed a similar trend (Fig. S4A and B). A total of 511 metabolites were differentially abundant across all treatments (Table S4). Of these, 182 (35.6% of the total) metabolites were uniquely differentially abundant only under the combined metal treatment. Interestingly, many of the differentially abundant metabolites (41.4%) were shared between the combined treatment and the Cu treatment alone. These trends were reflected in PCA results: the control and Ni-exposed metabolomes clustered together, while the Cu- and combined metal-exposed metabolomes were clearly separated from this cluster along PC1 (accounting for 34.2% of the variance in the negative ion mode metabolites) (Fig. S4C). Separation between the Cu-exposed and combined metal-exposed metabolomes was further observed along PC2 (20.6% of the variance). Similar results were observed whether considering the negative or positive ion mode metabolites (Fig. S4C and D).

Overall, these findings highlight a synergistic interaction at both the phenotypic level (Fig. 1B) and the molecular level (Fig. 2). We next analyzed the cellular systems that are differentially regulated during the combined Ni and Cu treatment to better understand (i) their mechanism of synergistic toxicity and (ii) the cellular acclimation to this complex form of stress.

### ROS are not involved in the synergistic toxicity of Ni and Cu

**Fig 3:**
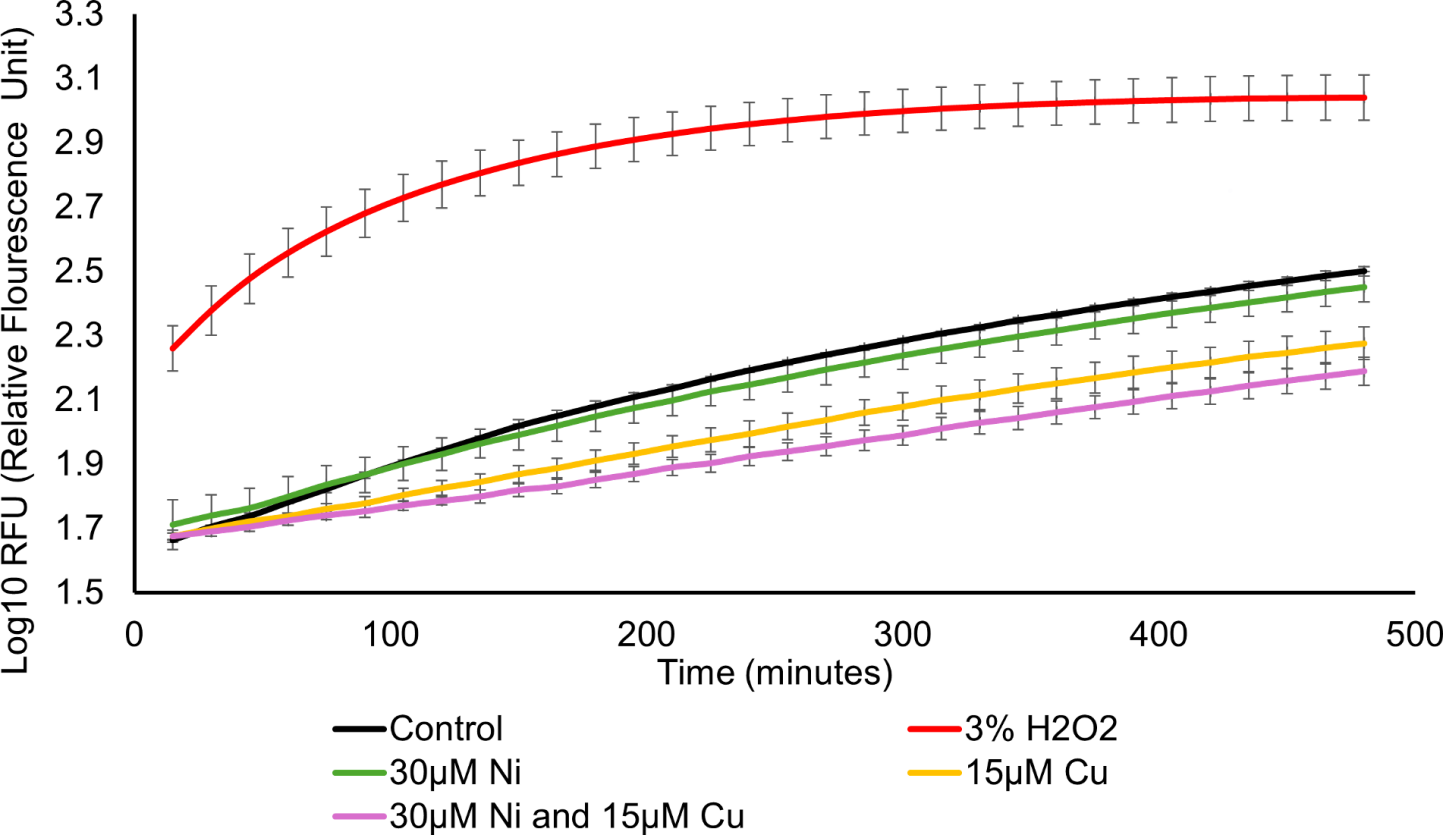
Measurement of intracellular ROS levels in *E. coli* following treatment with Ni, Cu, or combined metal stress. 3% hydrogen peroxide (H_2_O_2_) was used as a positive control. The “control” condition was untreated and serves as a negative control to establish the background level of fluorescence in the cultures. Each point represents the average of three replicates, and error bars represent ±SD. The y-axis values are presented as Log10-transformed data.

Both Ni and Cu have been proposed to exert their cytotoxic effects, at least partially, through ROS-dependent mechanisms. Cu generates superoxide and hydroxyl radicals via Fenton chemistry *in vitro* (55). By itself, Ni is a poor free radical generator (56); however, when complexed by ligands, such as cysteine (57), histidine (58), or oligopeptides (59), Ni also generates hydroxyl radicals *in vitro*. As our experiments were performed under aerobic growth conditions, we first considered whether ROS play a role in the synergistic toxicity of the two metals. However, we observed only a minimal transcriptional response from the “oxidative stress regulator” (OxyR) regulon under the three metal exposure conditions (60, 61) (Table S5). We also monitored intracellular ROS levels in metal-treated cultures with the probe 2′,7′-dichlorodihydrofluorescein diacetate (H_2_DCFDA). In its intracellular form, DCFH, the probe is responsive to hydrogen peroxide, hydroxyl radical, and peroxyl radical and is oxidized to the fluorophore DCF (62). The positive control (3% H₂O₂) caused an increase in ROS over time, detected by increasing fluorescence. However, treatment with Ni, Cu, or their combination did not result in a significant increase in fluorescence compared to the untreated negative control (Fig. 3). Interestingly, the production of ROS in the metal-exposed cultures was lower than the negative control, indicative of a possible suppression of basal ROS levels normally generated through respiratory processes at the electron transport chain (ETC). This suppression of basal ROS levels was minimal in Ni-treated cultures, moderate in Cu-treated cultures, and most pronounced in the combined treatment. We speculate that these data could indicate increasing damage to the ETC from the metal stress since we do not observe upregulation of antioxidant enzymes under our metal exposures (Table S5) (63). Alternatively, it could just be reflective of cell death and loss of ROS-generating biomass. Regardless of the cause of this ROS suppression, we concluded that other molecular mechanisms likely play a more prominent role in the synergistic toxicity of Ni and Cu.

### Ni and Cu stress dysregulates histidine metabolism

**Fig 4:**
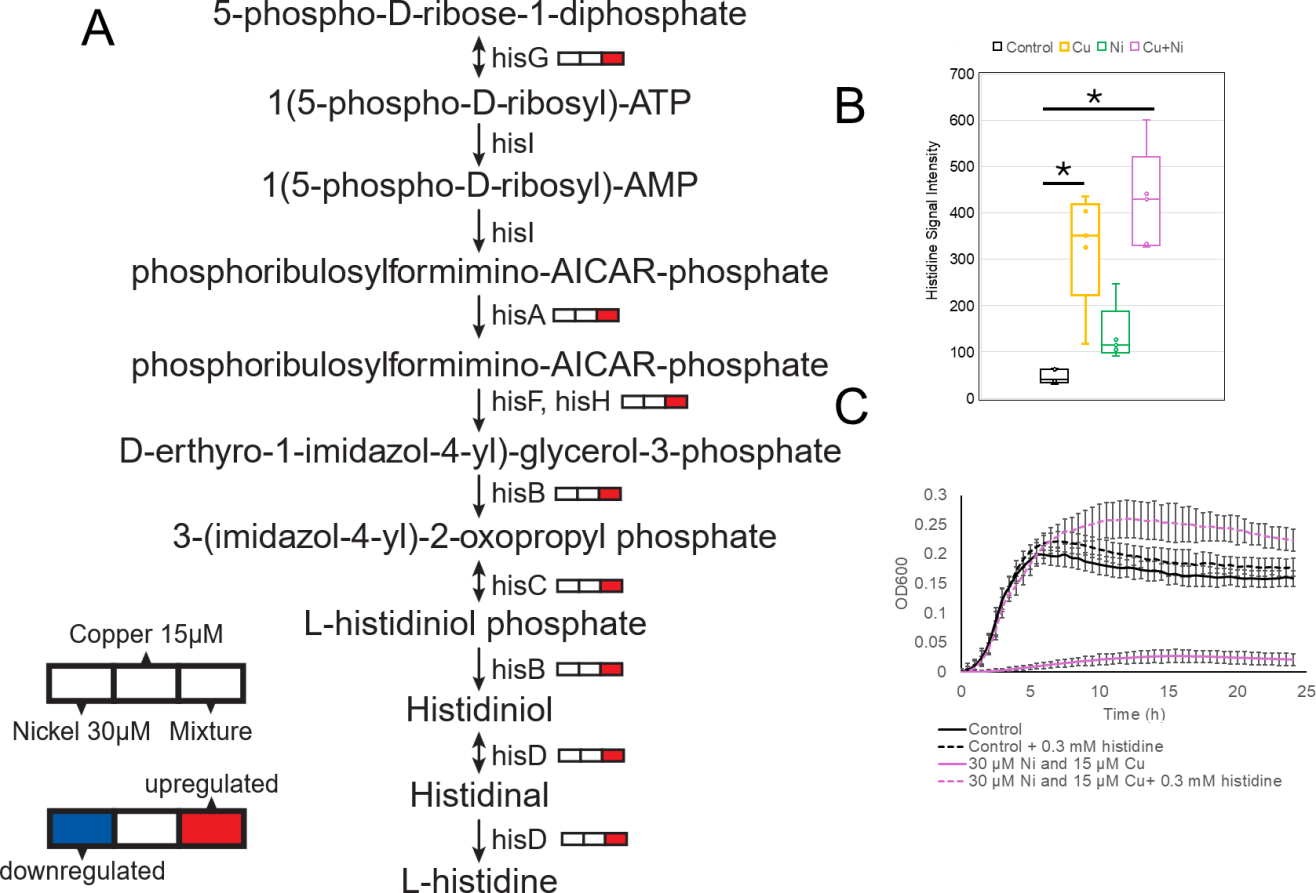
Histidine biosynthesis, metabolite levels, and functional rescue under metal stress. (**A**) Schematic of the histidine biosynthesis pathway in *E. coli*, highlighting genes that are differentially expressed following treatment with 30 µM Ni, 15 µM Cu, or a combination of both metals. (**B**) Intracellular histidine levels were measured by metabolomics after exposure to individual metals or the combined treatment, relative to the untreated control. * indicates *P* < 0.05. (**C**) Growth response of *E. coli* under combined metal stress (30 µM Ni +15 µM Cu) with or without 0.3  mM histidine supplementation, compared to controls with and without histidine. Each point represents the average of three replicates and error bars represent ±SD.

We observed upregulation of the *his* operon (except *hisI*) during the combined Ni and Cu exposure, but not during individual metal treatments (Fig. 4A). Metabolomics revealed elevated intracellular histidine concentrations during both the combined metal stress and Cu stress (Fig. 4B). The addition of histidine to the growth medium rescued the growth defect from the combined metal exposure (Fig. 4C) and eliminated the minor growth inhibition of the single metal exposures (Fig. S5). Histidine forms coordinate complexes with Ni^2+^ (64) as well as Cu^1+^ and Cu^2+^ (65, 66). With the exogenous histidine, extracellular metal chelation is likely occurring within the growth medium (i.e.*,* reducing the metal bioavailability [37, 67]), but the same chemistry is expected to occur intracellularly.

Transcription of the *his* operon is regulated by an attenuation mechanism that is sensitive to His-tRNA^His^ levels, which reflect intracellular histidine concentrations (68). We propose that histidine complexation with Ni and Cu depletes free histidine, derepressing the operon and enhancing histidine biosynthesis (69). Since no additional Ni or Cu is added to the system, the newly synthesized histidine accumulates within the cytosol (Fig. 4B). In Cu-only conditions, histidine accumulation still occurred without operon induction, suggesting that enzymatic feedback inhibition of HisG (ATP phosphoribosyltransferase) may be relieved by histidine binding to Cu (70). Complexation of intracellular histidine by Cu may be sufficient to relieve this feedback inhibition, but not enough to relieve the transcriptional attenuation of the *his* operon. A similar, though nonsignificant, trend was observed for the Ni-exposed cultures (Fig. 4B). We propose that the combined effect of Ni and Cu on histidine biosynthesis could be understood as the cumulative result of the complexation of both metals with intracellular histidine.

Given that exogenous histidine protects against the combined Ni and Cu toxicity, it was attractive to speculate that increased expression of the *his* operon and accumulation of intracellular histidine are important in the cellular response to this combined stress (71, 72). However, histidine biosynthesis mutant strains *ΔhisG* (ATP phosphoribosyltransferase) and *ΔhisD* (histidinol dehydrogenase) had similar growth phenotypes as the wild-type strain under all exposure conditions (Fig. S6), indicating that the observed biosynthetic changes are likely a chemical consequence of intracellular metal complexation, not an acclimatization response. This would still be consistent with prior studies that also reported increased expression of the *his* operon in other bacteria in response to high concentrations of either Ni or Cu (73–75). However, we note that the exposure durations and growth kinetics differed between our transcriptomic and metabolomic experiments (2-hour exposures during mid-log growth) and our mutant strain and amino acid supplementation experiments (≥24-hour exposures initiated from lag phase). Thus, it is possible that upregulation of histidine biosynthesis genes could provide a short-term benefit in actively growing cultures that is not apparent under the longer exposure regimes (76, 77) (Fig. S1).

### Modulation of sulfur metabolism in the response to combined Ni and Cu stress

**Fig 5:**
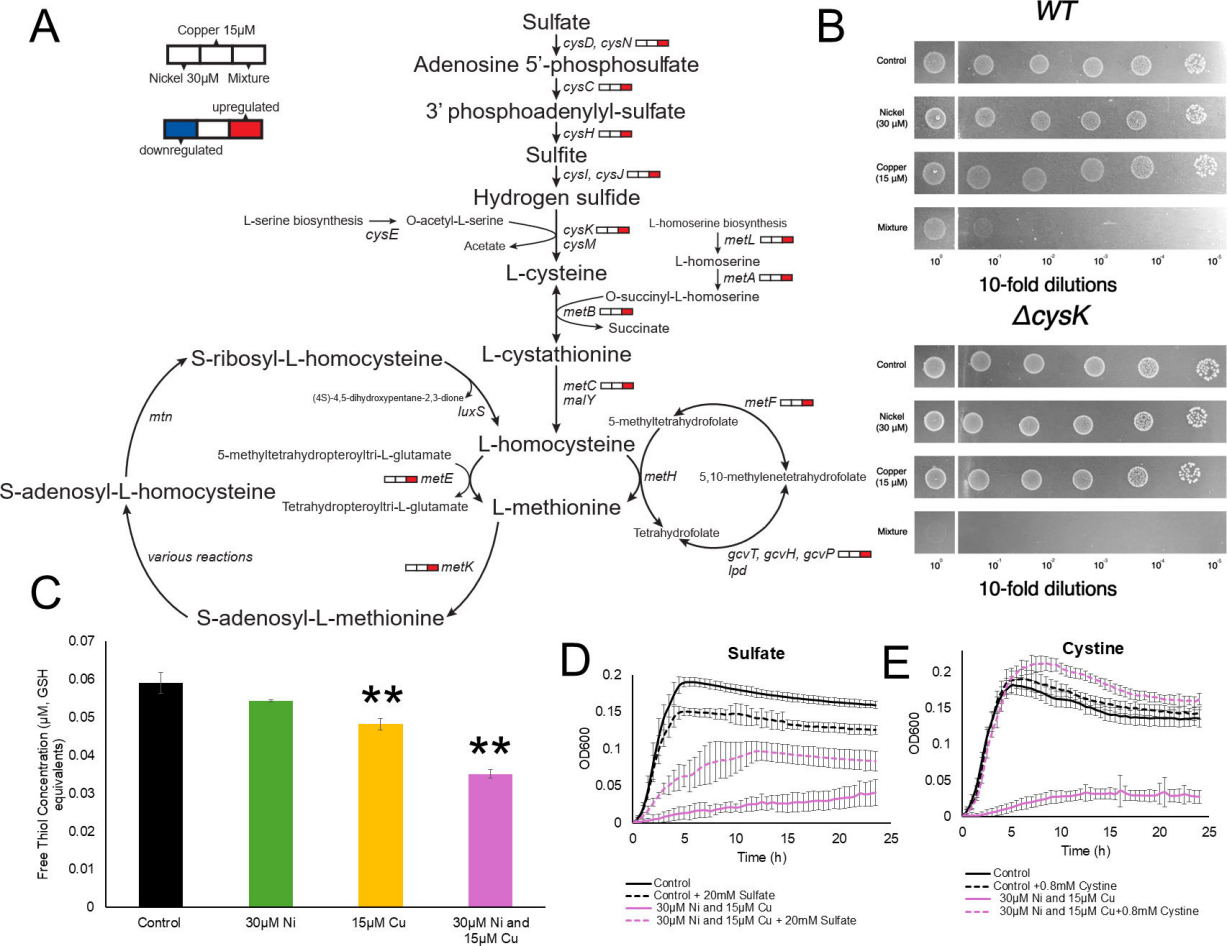
Sulfur assimilation, cysteine, and methionine metabolism are impacted by metal stress in *E. coli*. (**A**) Transcriptomic modulation of the sulfur assimilation and methionine biosynthesis pathways in *E. coli* during exposure to 30 µM Ni, 15 µM Cu, and their combination (Ni +Cu), relative to untreated controls. (**B**) Spot dilution assay comparing growth of *ΔcysK* mutant and wild-type *E. coli* under control conditions and metal treatments. Experiments were performed a minimum of two times, with one representative trial shown. Note that a single wild-type strain experiment is shown across figure panels, for ease of comparison, since experiments were performed in parallel. (**C**) Quantification of total intracellular thiol levels in cells exposed to Ni, Cu, and Ni + Cu treatments. **P  <  0.01; ***P  <  0.001. (**D**) Impacts of sulfate (20 mM) supplementation on growth under combined metal stress (30 µM Ni + 15  µM Cu). (**E**) Effect of 0.8 mM cystine supplementation on growth under combined metal stress. For panels C–E, each point represents the average of the replicates, and error bars represent ±SD.

Upon exposure to the combined Ni and Cu treatment, we observed significant upregulation of multiple genes involved in sulfate assimilation (Fig. 5A), including *cysN* (sulfate adenylyltransferase subunit 1), *cysC* (adenylyl-sulfate kinase), *cysD* (sulfate adenylyltransferase subunit 2), *cysH* (phosphoadenosine phosphosulfate reductase), *cysI* (sulfite reductase [NADPH] hemoprotein β-subunit)*, cysJ* (sulfite reductase [NADPH] flavoprotein α-subunit), and *cysK* (cysteine synthase A). This response did not occur with either the Ni or Cu treatments. Similarly, we observed the upregulation of *cysA* (ATP-binding component of the CysPUWA sulfate/thiosulfate ABC transporter) and *sbp* (sulfate/thiosulfate ABC transporter periplasmic-binding protein) under the combined Ni and Cu treatment only (Table S2) (78). This pathway is essential for assimilating inorganic sulfur into cysteine, a sulfur-containing amino acid (79). Consistent with our transcriptional data, supplementing the culture medium with 20 mM sulfate partially rescued growth during the combined metal stress (Fig. 5D). The addition of 20 mM sulfate to Ni-treated cultures enhanced growth, particularly during the early growth phase, whereas this same amount of sulfate reduced the final carrying capacity of the Cu-treated cultures (Fig. S7). However, since these effects are so minor, it is hard to draw any firm conclusions from these observations.

We hypothesized that this sulfur demand might stem from an increased need for cysteine and/or cysteine-derived compounds like glutathione or methionine during the combined metal stress. Indeed, we observed a decrease in the total intracellular thiol concentrations during the exposure to the combined metal stress (Fig. 5C). Although glutathione supplementation improved growth under the combined metal stress (and Cu stress) (Fig. S8), *gshA* (glutamate-cysteine ligase) and *gshB* (glutathione synthetase) (31) expression remained unchanged across all conditions (Table S2), and their deletion improved growth under the combined metal stress (Fig. S9). We speculate that glutathione amendments likely rescued growth, in part, through the extracellular chelation of the Cu^2+^ (80, 81).

Methionine biosynthesis genes were upregulated (Fig. 5A) and intracellular methionine levels increased under the combined metal stress, while levels of the pathway intermediate cystathionine decreased (Fig. S10A). Methionine supplementation (7 mM) also significantly rescued growth under the combined metal stress but did not affect growth under the single metal stressors (Fig. S10B). However, like glutathione, the methionine biosynthesis mutant strains *ΔmetA, ΔmetB,* and *ΔmetE* outperformed wild-type strain (Fig. S11), suggesting that methionine overproduction may be maladaptive during growth under the combined metal stress. However, as was the case with histidine, we note that future work will be needed to disentangle the role that methionine plays in the multi-metal stress response across different growth phases and exposure durations.

We next tested whether the sulfur limitation stress response was due to increased cysteine demand. We supplemented the metal-stressed cultures with 0.8 mM cystine, the disulfide form of cysteine that is reduced to cysteine intracellularly (82). Cystine completely alleviated the growth inhibition observed in the cultures treated with Ni and Cu, as well as Cu or Ni individually (Fig. 5E) (Fig. S12). In our transcriptome data, we noted the upregulation of genes encoding two cystine transporters during the combined metal stress: TcyP and the TcyJ subunit of the TcyJLN transporter (Table S2). Considering that we also observed increased expression of the cysteine synthase A gene (*cysK*) during the combined metal exposure (Fig. 5A), we next tested the growth of a *ΔcysK* mutant under our different exposure conditions. *ΔcysK* had comparable growth to the wild-type strain under the individual Cu or Ni exposures (Fig. 5B). In contrast, *ΔcysK* had weaker growth with the combined Cu and Ni exposure compared to the wild-type strain (Fig. 5B). Thus, cysteine biosynthesis is critical for survival under the combined metal stress. This is apparent both from our transcriptomic data, reflecting a more acute exposure of actively growing cells, and our supplementation and mutant growth experiments, which reflect longer exposures. Additionally, since our growth medium supplies amino acids in the form of yeast extract, we propose that the disruption of the methionine or glutathione biosynthesis pathways improved growth (Fig. S9 and S11) during the combined metal stress by rerouting cysteine to an unknown, growth-promoting pathway(s) active under these conditions.

### Iron-sulfur clusters are targeted by the combined metal stress

**Fig 6:**
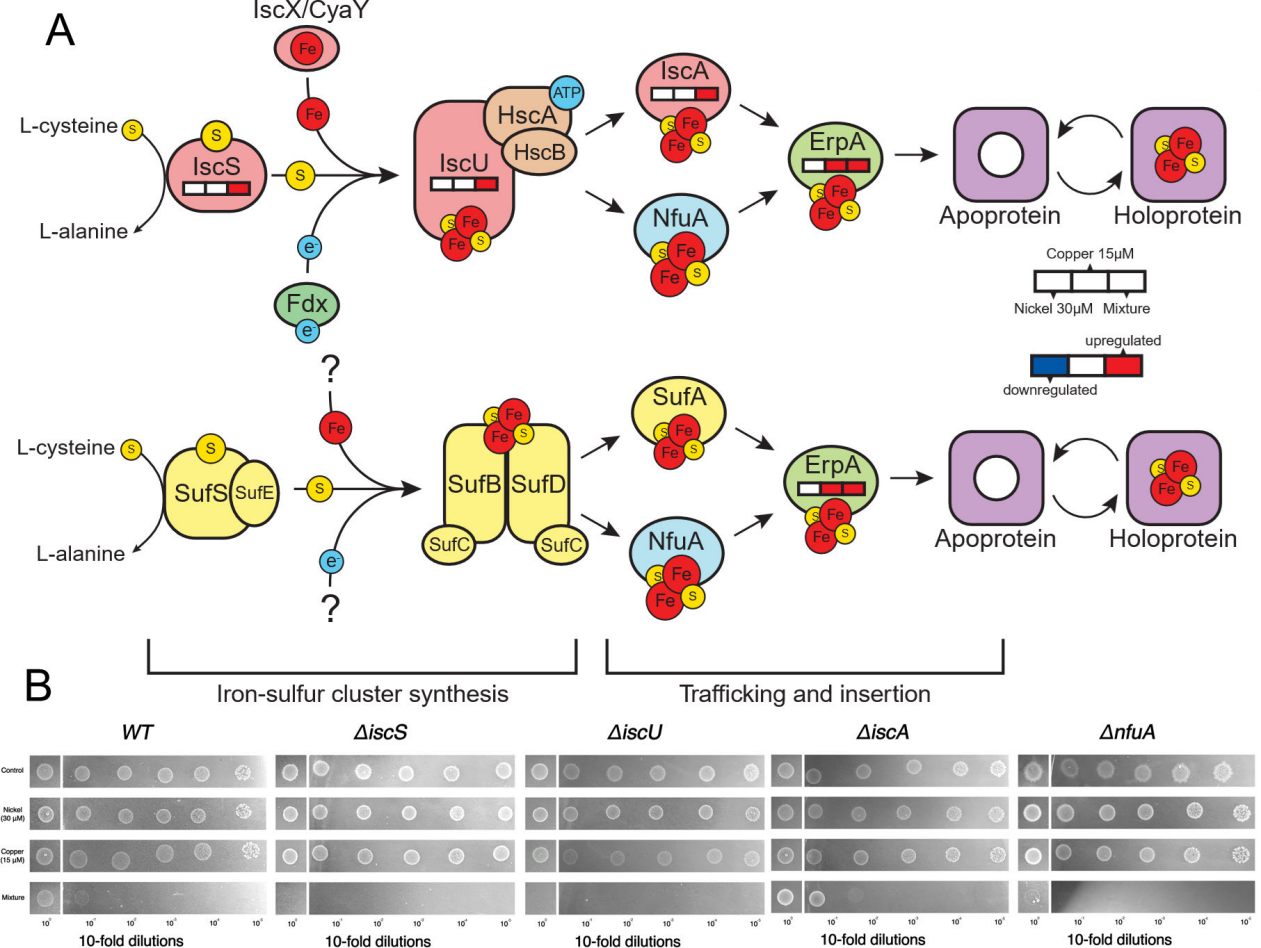
Iron-sulfur cluster biogenesis pathway and mutant phenotypes under metal stress conditions. (**A**) Schematic representation of the iron-sulfur (Fe-S) cluster synthesis pathway in *E. coli*, including its trafficking and insertion into target proteins. Genes involved in this pathway are overlaid with transcriptomic data showing differential expression in response to 30 µM Ni, 15 µM Cu, and combined Ni + Cu treatment. (**B**) Spot dilution assay showing the growth of wild-type *E. coli* and deletion mutants (*∆iscS, ∆iscU, ∆iscA*, and *∆nfuA*) under control and metal stress conditions. Serial 10-fold dilutions (10⁻^0^–10⁻⁵) were spotted on MBMM agar plates containing no metal (control), 30 µM Ni, 15 µM Cu, or both metals combined. Experiments were performed a minimum of two times, with one representative trial shown. Note that a single wild-type strain experiment is shown across figure panels, for ease of comparison, since experiments were performed in parallel.

One plausible sink for this cysteine is [Fe-S] cluster assembly since cysteine is the sulfur donor for [Fe-S] cluster biogenesis (2). Consistent with this model, we observed increased expression of genes involved in iron-sulfur [Fe-S] cluster assembly under combined metal stress (Fig. 6A). *E. coli* utilizes two main systems for Fe-S cluster biosynthesis: (i) the ISC (Iron-Sulfur Cluster) system, which functions as the primary housekeeping pathway and (ii) the SUF (Sulfur Utilization Factor) system, which is induced under stress conditions (83). Genes associated with the ISC system, including *iscS* (cysteine desulfurase), *iscU* (scaffold protein), and *iscA* (iron-sulfur cluster carrier protein), were upregulated in response to the metal mixture, but not under individual Ni or Cu treatments. In contrast, the SUF system genes were not differentially expressed under any condition. We also observed upregulation of *erpA* (Essential Respiratory Protein A) under the combined metal stress and Cu treatment. ErpA plays a crucial downstream role in delivering preassembled Fe-S clusters to apoprotein targets, particularly those involved in essential cellular processes like respiration and central metabolism (84).

 We next tested the growth of Fe-S cluster assembly and trafficking mutants with the different metal exposures. *ΔiscS* and *ΔiscU* strains were unable to grow under combined metal exposure, indicating that the core assembly machinery of the ISC system is essential under this condition (Fig. 6B). Notably, IscS has been previously linked to repair of damaged Fe-S clusters (85). In contrast, the *ΔiscA* mutant exhibited growth comparable to the wild type (Fig. 6B), consistent with the known redundancy of auxiliary ISC components (86–88). *ΔnfuA* had weaker growth with the metal combination relative to the wild-type strain (Fig. 6B). This aligns with recent findings that NfuA interacts with ErpA to transfer Fe-S clusters from the ISC and SUF scaffolds to essential respiratory and other metabolic proteins (89, 90). *erpA* itself is essential in *E. coli* and cannot be deleted (89). We also confirmed that the SUF system was not required for growth under the combined metal stress. Under the combined metal stress, both *ΔsufD* and *ΔsufS* had, surprisingly, enhanced growth while *ΔsufA* had similar growth relative to the wild-type strain (Fig. S13). None of the mutant strains had altered growth under any of the other conditions tested (Fig. 6B; Fig. S13). To our knowledge, improved growth of *suf* mutants has not yet been reported in *E. coli,* one of the few model microorganisms to have dual Fe-S biogenesis systems (91). *E. coli suf* mutants typically have wild-type levels of Fe-S cluster enzyme activity (85). Likewise, SUF is not required for Fe-S cluster repair, which is dependent on IscS (85). However, recent work by Bak and Weerapana (92) hinted at the possibility of some distinct client proteins of the *E. coli* SUF and ISC systems. If this is true, disabling SUF may shift resources to ISC-dependent Fe-S biogenesis for enzymes that are essential under the growth conditions used here. However, further work is required to investigate this hypothesis.

The observation that SUF was not essential for growth under the combined metal stress was surprising, as the SUF system is typically induced under stress conditions where the ISC system is impaired, specifically oxidative damage or iron (Fe) limitation (93). However, this finding was consistent with our earlier observation that intracellular ROS levels did not increase significantly under any metal exposure (Fig. 3). Fe^2+^ supplementation also failed to rescue growth (Fig. S14), nor did we observe significant de-repression of the Fur regulon (94) under the combined metal exposure. In fact, expression of the *feoABC* operon, encoding the Fe^2+^ transporter, was decreased during the combined metal exposure (95) (Table S2). Thus, sulfur—rather than Fe—appears to be the limiting nutrient for Fe-S biosynthesis under combined metal stress. This contrasts with our prior findings in *B. cereus*, where a more complex metal mixture led to a broad Fe starvation response (30).

The *iscRSUA-hscBA-fdx (isc*) operon and *erpA* are both regulated by the transcription factor IscR. When bound to a [2Fe-2S] cluster, IscR represses the expression of these genes (96). Prior studies in *E. coli* suggest that loss of the bound [2Fe-2S] leads to de-repression of these genes and occurs through two mechanisms: (i) decreased synthesis of Fe-S clusters (97) and (ii) damage to extant Fe-S clusters (98). We propose a model where both might occur during the combined metal stress, leading to increased expression of the ISC system as well as *erpA* (Fig. 6A). The divalent metal cations cobalt (Co^2+^), zinc (Zn^2+^), and cadmium (Cd^2+^) bind to the active site of the *E. coli* IscU scaffold protein (99), whereas Cu^1+^ does not bind to IscU (100). This selectivity is governed by the coordination geometry preferences of the IscU active site (99). It is conceivable that Ni^2+^, with its similar coordination chemistry to Co^2+^ (101), selectively binds to IscU over Cu^1+^. Furthermore, due to its placement within the Irving-Williams series, Ni^2+^ would also out-compete cellular Fe^2+^ for IscU binding (102). Binding of IscU to transition metals other than Fe^2+^ deactivates the enzyme, inhibiting *de novo* Fe-S cluster biosynthesis (103, 104). While Cu^1+^ does not bind IscU, Cu^1+^ does damage existing Fe-S clusters by directly displacing Fe through interaction with coordinating cysteine thiolates, deactivating Fe-S cluster-containing enzymes (105). In contrast, *in vitro* experiments have suggested that Ni^2+^ does not directly alter the activity of Fe-S cluster-containing enzymes (106).

According to our model, during Cu exposure, Fe-S cluster degradation can be countered by the maturation of new Fe-S by a functional ISC system. We see some evidence for this in our transcriptome data with increased expression of *erpA* during Cu exposure (Fig. 6A). During Ni exposure, maturation of new Fe-S clusters is less important if there is minimal damage to existing clusters—supported by the lack of transcriptional changes related to Fe-S cluster biosynthesis under these conditions. We propose that the two metals synergize in their toxicity by (i) damaging the existing Fe-S clusters within enzymes and then (ii) inhibiting biosynthesis of new clusters. This model provides further context to our observation that sulfur is the limiting nutrient for Fe-S biosynthesis under combined metal stress, rather than Fe. Metal-induced damage to Fe-S clusters increases intracellular Fe^2+^ concentrations (106–108), which would explain the decreased expression of the Fur-regulated *feoABC* during both the combined metal stress and the individual Cu stress (Table S2). Dysregulation of Fe-S cluster metabolism by the combined metal stress also puts a significant strain on cellular sulfur metabolism in two ways. First, as we noted earlier, cysteine is the sulfur donor for ISC (83). Second, assimilatory sulfate reduction to cysteine is dependent upon Fe-S cluster metabolism, as the sulfite reductase (CysJI) has multiple Fe-S cluster cofactors. Damage to Fe-S clusters is known to reduce sulfite reductase activity (109). This would explain our observation that high concentrations of sulfate (20 mM) only partially rescue growth during the combined metal stress, while cystine at a lower concentration (0.8 mM cystine or 1.6 mM cysteine) fully rescues growth under the same condition (Fig. 5D and E). Decreased metabolic flux through the sulfur assimilation pathway may also have caused the increase in expression of the methionine biosynthesis pathway under the combined metal stress (Fig. 5A). In *Salmonella enterica,* it was reported that Co^2+^ damages Fe-S clusters, leading to decreased sulfite reductase activity, which resulted in a methionine auxotrophic requirement (109, 110).

### Reprogramming of the tricarboxylic acid cycle

**Fig 7:**
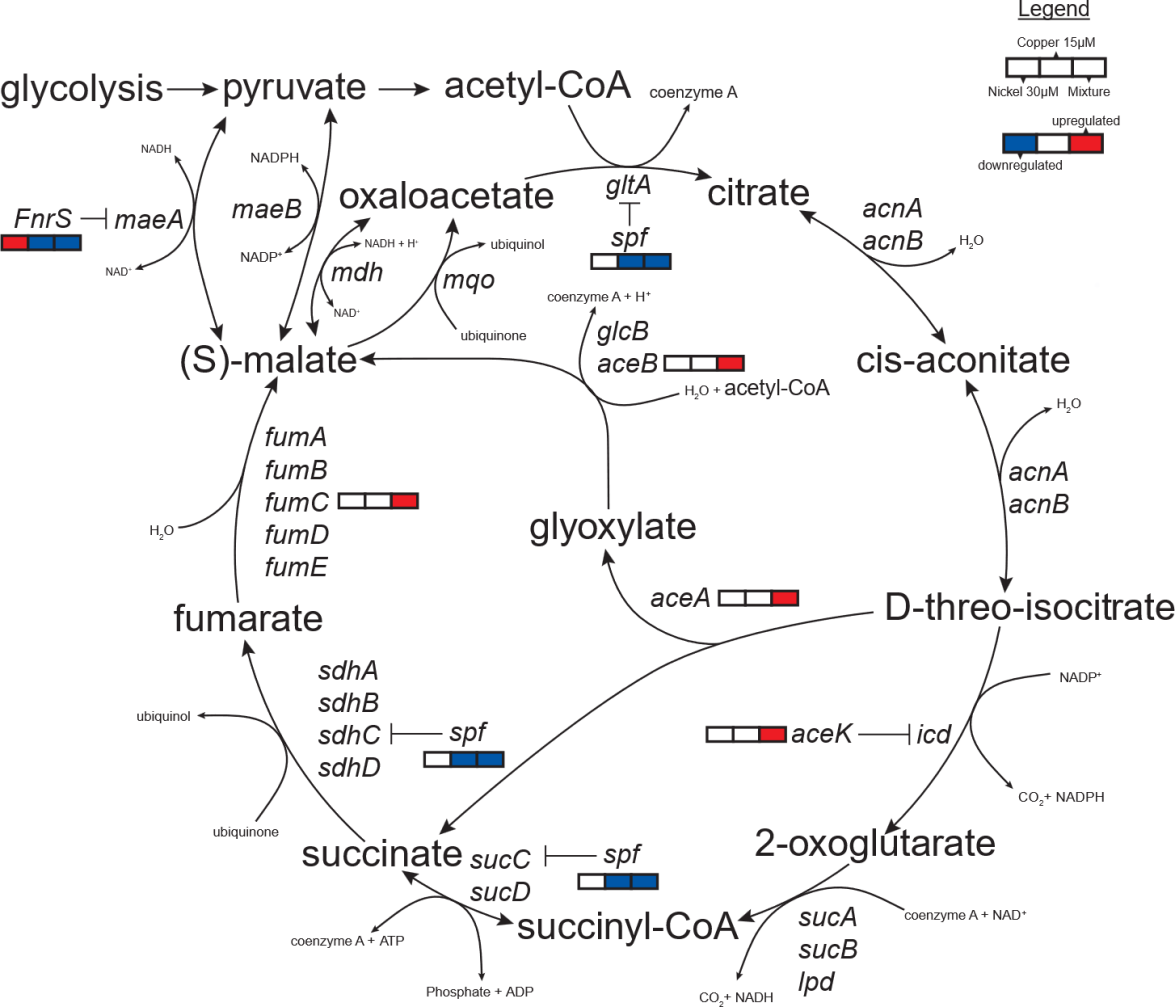
Changes in the expression of genes encoding TCA cycle and glyoxylate shunt enzymes and small RNAs (*fnrS* and *spf*) during the metal exposures. Genes involved in this pathway are overlaid with transcriptomic data showing differential expression in response to 30 µM Ni, 15 µM Cu, and the combined Ni + Cu treatment.

Fe-S clusters have essential roles in electron transfer, central metabolism, and gene regulation, making them critical to cellular functioning (2). Therefore, we considered the downstream consequences of Fe-S cluster disruption under combined metal stress. Transcriptomic analysis revealed modulation of the tricarboxylic acid (TCA) cycle and related pathways only under the combined metal treatment (Fig. 7). Several enzymes of the TCA cycle (i.e., aconitase [111], succinate dehydrogenase [112], fumarase [113]) are dependent on Fe-S clusters for their activities. Accordingly, *fumC* (fumarase C), encoding an alternative, [Fe-S]-independent fumarase (113), was upregulated only in the combined Ni and Cu condition (Fig. 7). The small RNA *spf* (small regulatory RNA Spot 42), which inhibits the translation of key TCA cycle genes—*gltA* (citrate synthase) and *sdhCDAB* (succinate dehydrogenase) (Fig. 7) (114–116)—was also downregulated under both Cu and combined metals treatments. Decreased expression of *spf* would relieve translational repression of key TCA cycle enzymes, promoting sustained flux through the TCA cycle to support energetic and biosynthetic demands under combined metal stress.

Genes of the glyoxylate shunt—*aceA* (isocitrate lyase), *aceB* (malate synthase A), and *aceK* (isocitrate dehydrogenase kinase/isocitrate dehydrogenase phosphatase)—were significantly upregulated only under the combined metal stress (Fig. 7). This shunt bypasses the CO₂-releasing steps of the TCA cycle, allowing *E. coli* to conserve carbon and redirect intermediates toward anabolic pathways (117–119). AceA performs the first step of the shunt, the cleavage of isocitrate to succinate and glyoxylate, while AceB converts glyoxylate to malate. AceK is the regulatory switch that turns on or off the activity of isocitrate dehydrogenase, controlling the partitioning of carbon between the TCA cycle and glyoxylate shunt. While glyoxylate concentrations were increased during the combined metal exposure, they did not reach the level of significance (Fig. S15). Malate appears to be maintained at a low level in the cells, as it was not detected under any condition. Succinic acid was significantly increased during the combined metal exposure (Fig. S15). This could be attributed to the increased flux through the glyoxylate shunt, decreased activity of the Fe-S-cluster enzyme succinate dehydrogenase (112), or a combination of the two.

The glyoxylate shunt has been implicated in the response to stressors, including hypoxia (120), ROS (119, 121), antibiotics (122, 123), and Fe limitation (124, 125). During Fe limitation, shifting carbon flux through the glyoxylate shunt allows cells to bypass the steps of the TCA cycle reliant upon the heavily Fe-cofactor-dependent ETC. The same logic could be applied to multi-metal stress, as Complex I of the *E. coli* aerobic ETC, required for NADH re-oxidation, has multiple Fe-S cluster cofactors. However, it is also possible that conservation of carbon via the glyoxylate shunt is required to support the *de novo* synthesis of amino acids like histidine, cysteine, and methionine, whose biosynthetic pathways are upregulated during the combined metal exposure (Fig. 4 and 5A). We next tested whether this shift from the full TCA cycle to the glyoxylate shunt is adaptive by examining the growth of the mutant strains *ΔaceB, ΔaceA,* and *ΔaceK* under the different metal exposures (Fig. S16). *ΔaceB* and *ΔaceA* had similar phenotypes to the wild-type strain, while *ΔaceK* had a more severe growth defect under the combined metal stress. Under all other conditions, the three mutant strains grew similarly to the wild-type strain. These data suggest that the regulatory switch between the full TCA cycle and the glyoxylate shunt by AceK is critical in the cellular response to the metal mixture stress.

The post-transcriptional regulatory small RNA *fnrS* showed condition-specific expression: it was downregulated under Cu stress and the combined metal exposures but upregulated during Ni exposure alone. One *fnrS* target, *maeA* (malate dehydrogenase), links the TCA cycle with pyruvate metabolism (Fig. 7) (126, 127). Reduced *fnrS* under combined stress may increase *maeA* translation, boosting malate-to-pyruvate flux and enhancing carbon routing flexibility. *fnrS* is upregulated by Fnr (128), a global regulator of the aerobic-anaerobic transition that is itself activated by a [4Fe-4S] cluster (129, 130). This cluster is sensitive to damage from oxygen (131) or nitric oxide (132)—and perhaps also from Cu during the combined metal stress. While our experiments were performed under aerobic growth conditions, during mid-to-late exponential growth—the period captured by our transcriptome experiments—oxygen typically becomes depleted over time in shaken flask cultures of *E. coli*, leading to increased expression of the Fnr regulon (98, 133). Indeed, we see that the control, Ni-, and Cu-exposed cells continue to grow (Fig. S1)**,** while the cells exposed to the two metals in combination immediately cease growth following supplementation. The three actively growing cultures likely continued to deplete oxygen from the medium, which would initiate the aerobic-to-anaerobic transition—a process that appears to be disrupted when Cu is present. We propose that this disruption may result from damage to the Fnr [4Fe-4S] cluster by Cu, inhibition of oxygen consumption from the culture medium by the combined metal stress (potentially through ETC impairment or cell death), or a combination of both.

Consistent with Fnr inactivation, multiple Fnr-regulated genes showed reduced expression under combined metal stress, including *dmsA* (DMSO reductase subunit A)*, nikC* (nickel ABC transporter membrane subunit)*, yfiD* (stress-induced alternate pyruvate formate-lyase)*, narGHIJ* (respiratory nitrate reductase)*, ydfZ* (selenoprotein YdfZ)*, nirBDC* (nitrite reductase)*, nrfA* (assimilatory nitrate reductase)*, caiF* (transcriptional activator CaiF)*, narK* (nitrate:nitrite antiporter), *frdABC* (fumarate reductase)*, hcp* (nitric oxide reductase)*, dcuB* (anaerobic C4 dicarboxylate transporter)*, dcuC* (anaerobic C4 dicarboxylate transporter), *ynfK* (dethiobiotin synthetase)*,* and *yjiM* (dehydratase) (134). Many of these same genes were also downregulated during Cu exposure (Table S2). These expression patterns support a model where, during combined metal stress, the Cu damages the Fnr cluster, repressing the Fnr regulon.

Altogether, these transcriptional changes suggest that co-exposure to Ni and Cu induces a broad metabolic reprogramming involving both enhancement of key TCA cycle functions, activation of the glyoxylate shunt, and inhibition of pathways involved in adaptation to anaerobiosis. We speculate that many of these observed changes likely link back to dysregulation of Fe-S cluster metabolism; however, future work is required to confirm this model.

### Conclusions and environmental significance

Freshwater concentrations of Cu are typically below (21, 48) regulatory thresholds set by the World Health Organization (31 µM) and the US Environmental Protection Agency (USEPA 20 µM) (135). For example, a recent survey of 179 freshwater bodies found that only three sites exceeded this USEPA limit (21), with a median Cu concentration of 0.11 µM. Even in contaminated environments, Cu concentrations often do not surpass action levels. For instance, in an aquifer impacted by improper heavy metal waste disposal, Cu concentrations were reported at 0.005–15 µM (48). In a stream near a Cu mine, Cu concentrations were no higher than 16 µM (136) However, Cu rarely exists in isolation from other metals (21). It frequently co-occurs with other metals such as Ni (Fig. 1A).

We propose that Ni, whose concentrations are positively correlated with Cu concentrations across environmental data sets (Fig. 1A)**,** amplifies Cu toxicity in bacteria by potentiating Cu-induced disruption of Fe-S cluster metabolism. Indeed, we propose that disruption of Fe-S clusters is likely the primary mechanism of toxicity in cells exposed to Ni and Cu in combination. This is supported by both our transcriptional data (representing a shorter-term exposure of actively growing cells) and our mutant fitness data (representing a longer exposure from lag phase) where disruption of *isc* and *nfuA* genes eliminates growth under the combined metal stress (Fig. 6). Fe-S clusters are central to all forms of microbial metabolism, including the generation of biosynthetic precursors (112, 137), amino acid biosynthesis (105, 138), aerobic respiration (139), nitrate respiration (140), sulfate respiration (141), fermentation (142), nitrogen fixation (143), methanogenesis (144), acetogenesis (145), and carbon fixation (146–148). While our study uses *E. coli* as a model, this vulnerability likely extends across diverse prokaryotic and eukaryotic microbes. Understanding the synergistic toxicity of co-occurring metals like Ni and Cu is critical for accurately predicting microbial responses to metal pollution, a growing threat to ecosystem function in the Anthropocene (149).

## Data Availability

Raw reads for the transcriptomic data are deposited in the Sequence Read Archive under accession numbers SRR34735813–SRR34735824.

## SUPPLEMENTAL MATERIAL

Supplemental Material aem.01627-25-s0001.docx Supplemental methods, Tables S1 and S5, and Fig. S1 to S16.

Table S2 aem.01627-25-s0002.csv Combined transcriptomics data sets.

Table S3 aem.01627-25-s0003.csv TPM data for replicates.

Table S4 aem.01627-25-s0004.xlsx Metabolomics data.

ASM does not own the copyrights to Supplemental Material that may be linked to, or accessed through, an article. The authors have granted ASM a non-exclusive, world-wide license to publish the Supplemental Material files. Please contact the corresponding author directly for reuse.
