# Supplementary material for: The molecular basis of the synergistic toxicity of nickel and copper, common environmental co-contaminants
Source: Appl Environ Microbiol. 2025 Nov 24;91(12):e01627-25. doi: 10.1128/aem.01627-25 (PMC12724342; doi:10.1128/aem.01627-25)
Supplement: Supplemental Material — Supplemental methods, Tables S1 and S5, and Fig. S1 to S16. [file aem.01627-25-s0001.docx]

**SUPPORTING INFORMATION FOR**

**The molecular basis of the synergistic toxicity of Ni and Cu, common environmental co-contaminants**

^1^Linda Darwiche, ^2^Carlos A. Rodriguez-Bornot, ^2^Rebecca A. Ingrassia, ^3^Max J. Loccisano, ^4^Gray Waldschmidt, ^1,*^Jennifer L. Goff

^1^Department of Chemistry, SUNY College of Environmental Science and Forestry, Syracuse, NY, USA 13210

^2^Department of Environmental Biology, SUNY College of Environmental Science and Forestry, Syracuse, NY, USA 13210

^3^Division of Environmental Science, SUNY College of Environmental Science and Forestry, Syracuse, NY, USA 13210

^4^Department of Sustainable Resources Management, SUNY College of Environmental Science and Forestry, Syracuse, NY, USA 13210

*Corresponding author: [jegoff@esf.edu](mailto:jegoff@esf.edu)

**SUPPLEMENTAL METHODS**

**Media Preparation:**  To prepare 1 L of MBMM, 1.95 g of (N-morpholino) ethanesulfonic acid (MES)(Gold Bio, USA), 10 mg of sodium phosphate dibasic anhydrous (Na_2_HPO_4_)(VWR, USA), 50 mg of ammonium chloride (NH_4_Cl)(VWR, USA), 0.14 g of calcium sulfate (CaSO_4_)(Carolina Biological Supply, USA), 0.24 g of magnesium sulfate heptahydrate (MgSO_4_·7H_2_O)(VWR, USA), 20 mg of potassium chloride (KCl)(Acros Organics B.V.B.A, USA), 4 mg of iron sulfate heptahydrate (FeSO_4_·7H_2_O)(Fisher Scientific, USA), 1 mL of SL7 trace element solution (1000X), 10 mL of glucose (20% w/v)(VWR, USA), 0.5 g of yeast extract (MP Biomedicals, USA) were added to 990 mL of distilled water (DI H_2_O). The pH was adjusted to 6.4 using HCl or sodium hydroxide pellets, and the solution was sterilized by autoclaving. For the preparation of the SL7 trace element solution, 70 mg of zinc chloride (ZnCl_2_) (Fisher Scientific, USA), 100 mg of manganese chloride tetrahydrate (MnCl_2_·4H_2_O) (Carolina, USA), 60 mg of boric acid (H_3_BO_3_) (BTC, EU), 200 mg of cobalt chloride hexahydrate (CoCl_2_·6H_2_O)(Fisher Scientific, USA), 20 mg of copper chloride dihydrate (CuCl_2_·2H_2_O) (Fisher Scientific, USA), 20 mg of nickel chloride hexahydrate (NiCl_2_·6H_2_O) (MP Bio Medicals, USA), and 40 mg of sodium molybdate dihydrate (NaMoO_4_·2H_2_O) (BTC, EU) were dissolved in 1 L of water.

**RNA Sequencing:** Library preparation and RNA sequencing were performed at Biomarker Technologies (BMKGENE) USA Inc. (Durham, NC, USA). Samples were shipped frozen on dry ice. For library preparation, the Ribo-off rRNA Depletion Kit V2 (Bacteria) (N417, Vazyme, Nanjing, China) was used for rRNA depletion. Then, the ALFA-SEQ Directional RNALibPrepKit (RL1020, Magigene, Guangzhou, China) was used, following the manufacturer’s recommendations. Library quality control was performed using the QSep-400 (BiOptic, Taipei, China) and Qubit 3.0 (Thermo Fisher Scientific, Wilmington, USA). The library was sequenced on the Illumina Novaseq platform (Illumina, San Diego, USA) with paired-end 150 bp (PE150) mode.

**LC-MS Metabolomics:**  The liquid chromatography-mass spectrometry (LC-MS)-based untargeted metabolomic analysis was performed at Creative Proteomics (Shirley, NY, USA). Samples were shipped frozen on dry ice. For metabolite extraction, all samples were lyophilized and treated with 500 μL of 80% methanol. Each sample was vortexed, followed by sonication for 30 minutes at 4°C. Each sample was then incubated at -20°C for one hour, followed by vortexing for 30 seconds and then held at 4°C for 15 minutes. Samples were then centrifuged at 12,000 rpm and 4°C for 10 minutes. Finally, 200 μL of supernatant and 5 μL of DL-o-Chlorophenylalanine (0.14 mg/mL) was transferred to vial for LC-MS analysis.

The separations were performed using a Vanquish Flex UPLC combined with Q Exactive plus (Thermo) and detected by electrospray ionization mass spectrometry (ESI-MS). The LC system was equipped with an ACQUITY UPLC HSS T3 column (100×2.1mm×1.8 μm). The mobile phase was composed of solvent A (0.05% formic acid in water) and solvent B (100% acetonitrile) with a gradient elution (from 0 to 1 minutes held at 5% B; from 1 to 12 minutes ramping from 5% to 95% B; from 12 to 13.5 minutes holding at 95% B; from 13.5 to 13.6 min going from 95% to 5% B; and from 13.6 to 16 min held at 5% B. The flow rate of the mobile phase is 0.3 mL·min^-1^. The column temperature is maintained at 40°C, and the sample manager temperature is set at 4°C. Mass spectra were collected in both ESI+ (Heater Temp 300°C; Sheath Gas Flow rate, 45 arb; Aux Gas Flow Rate, 15 arb; Sweep Gas Flow Rate, 1 arb; spray voltage, 3.0 KV; Capillary Temp, 350°C; S-Lens RF Level, 30%) and ESI- (Heater Temp 300°C, Sheath Gas Flow rate, 45 arb; Aux Gas Flow Rate, 15 arb; Sweep Gas Flow Rate, 1 arb; spray voltage, 3.2 KV; Capillary Temp,350°C; S-Lens RF Level, 60%). modes. All mass spectra were acquired under both positive and negative modes. Data were normalized by dividing the peak area of each metabolite by the sum of all metabolites area and then multiplying by one million. The identification of metabolites was performed through automatic identification by Compound Discoverer (Thermo Scientific) and manual screening with the settings: Δm < 30 ppm and isotope similarity > 80%.

In untargeted metabolomics, data are acquired in both positive and negative ionization modes to maximize metabolite coverage. Some metabolites are detected in both modes due to their ability to ionize under both conditions, resulting in redundancy across datasets. In our data analysis, when a metabolite was detected in both modes and we needed to compare its abundance across conditions, we retained the entry with the lower adjusted p-value to prioritize the more statistically significant measurement. This is highlighted in red in the main figure.

**Table S1. *Escherichia coli* K-12 BW25113 Parent Strain Description**

| **Strain** | **Keio Collection Strain ID** | **Genotype** | **Source** |
| --- | --- | --- | --- |
| *Escherichia coli* K-12 BW25113 | n/a | F⁻, Δ(araD-araB)567, ΔlacZ4787(::rrnB-3), λ⁻, rph-1, Δ(rhaD-rhaB)568, hsdR514 | Horizon Discovery, Datsenko and Wanner (2000) |
| *ΔmetA* | JW3973 | BW25113 ΔmetA::kan | Horizon Discovery, Baba et al. (2006) |
| *ΔmetB* | JW3910 | BW25113 ΔmetB::kan | Horizon Discovery, Baba et al. (2006) |
| *ΔmetC* | JW2975 | BW25113 ΔmetC::kan | Horizon Discovery, Baba et al. (2006) |
| *ΔmetE* | JW3805 | BW25113 ΔmetE::kan | Horizon Discovery, Baba et al. (2006) |
| *ΔmetI* | JW0194 | BW25113 ΔmetI::kan | Horizon Discovery, Baba et al. (2006) |
| *ΔcysA* | JW2415 | BW25113 ΔcysA::kan | Horizon Discovery, Baba et al. (2006) |
| *ΔcysB* | JW1267 | BW25113 ΔcysB::kan | Horizon Discovery, Baba et al. (2006) |
| *ΔcysN* | JW2721 | BW25113 ΔcysN::kan | Horizon Discovery, Baba et al. (2006) |
| *ΔcysK* | JW2407 | BW25113 ΔcysK::kan | Horizon Discovery, Baba et al. (2006) |
| *ΔgshA* | JW2663 | BW25113 ΔgshA::kan | Horizon Discovery, Baba et al. (2006) |
| *ΔgshB* | JW2914 | BW25113 ΔgshB::kan | Horizon Discovery, Baba et al. (2006) |
| *ΔiscS* | JW2514 | BW25113 ΔiscS::kan | Horizon Discovery, Baba et al. (2006) |
| *ΔiscU* | JW2513 | BW25113 ΔiscU::kan | Horizon Discovery, Baba et al. (2006) |
| *ΔiscA* | JW2512 | BW25113 ΔiscA::kan | Horizon Discovery, Baba et al. (2006) |
| *ΔsufD* | JW1671 | BW25113 ΔsufD::kan | Horizon Discovery, Baba et al. (2006) |
| *ΔsufS* | JW1670 | BW25113 ΔsufS::kan | Horizon Discovery, Baba et al. (2006) |
| *ΔsufA* | JW1674 | BW25113 ΔsufA::kan | Horizon Discovery, Baba et al. (2006) |
| *Δmrp* | JW2100 | BW25113 Δmrp::kan | Horizon Discovery, Baba et al. (2006) |
| *ΔnfuA* | JW3377 | BW25113 ΔnfuA::kan | Horizon Discovery, Baba et al. (2006) |
| *ΔhisD* | JW2002 | BW25113 ΔhisD::kan | Horizon Discovery, Baba et al. (2006) |
| *ΔhisG* | JW2001 | BW25113 ΔhisG::kan | Horizon Discovery, Baba et al. (2006) |
| *ΔaceA* | JW3975 | BW25113 ΔaceA::kan | Horizon Discovery, Baba et al. (2006) |
| *ΔaceB* | JW3974 | BW25113 ΔaceB::kan | Horizon Discovery, Baba et al. (2006) |
| *ΔaceK* | JW3976 | BW25113 ΔaceK::kan | Horizon Discovery, Baba et al. (2006) |


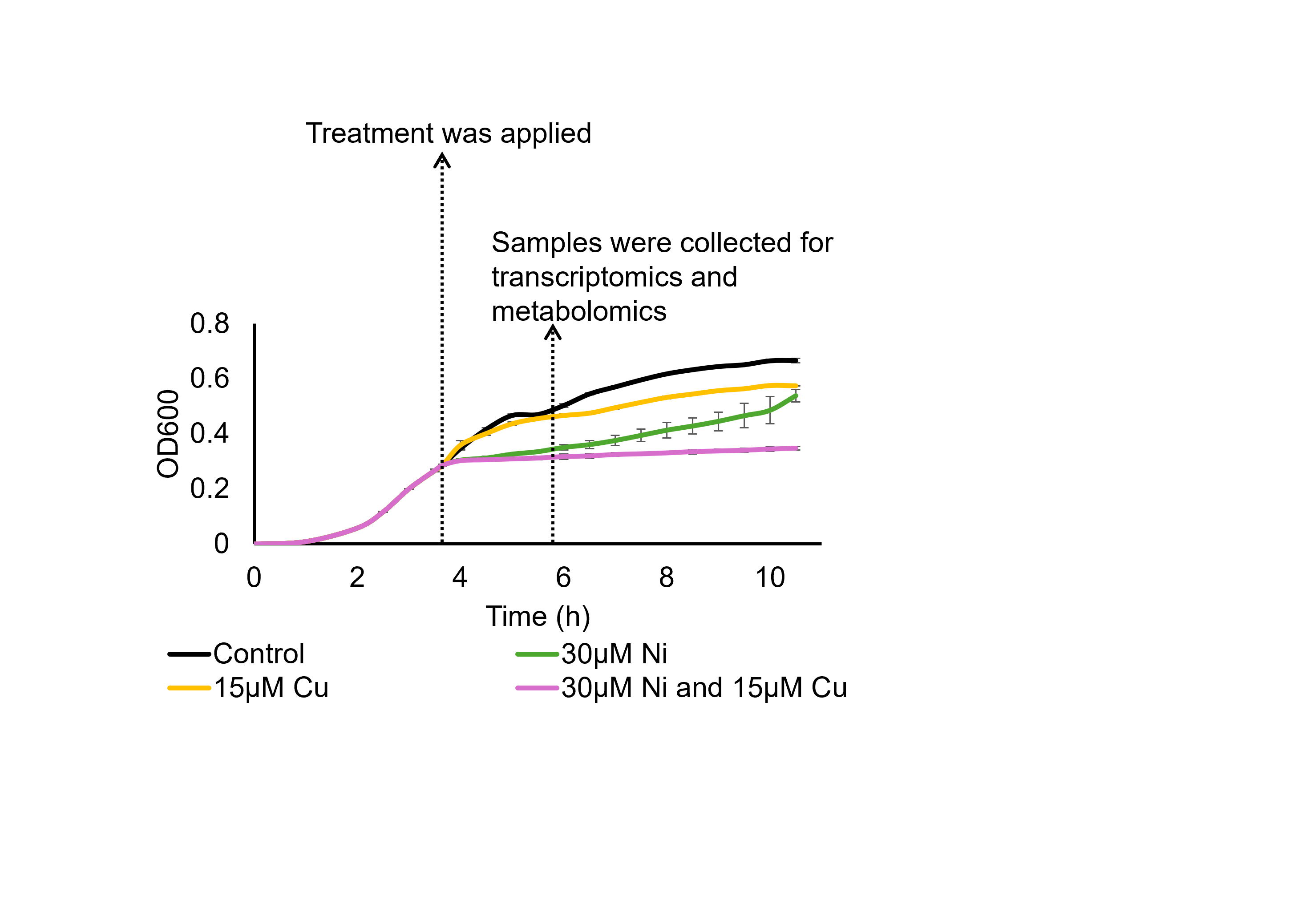


**Figure S1.** Growth curves of E. coli under the conditions used for collecting samples for transcriptomic and metabolomic analyses. Cultures were allowed to grow to mid-log phase. At this point, one of four treatments was applied: (1) a control (no treatment), (2) 30 μM Ni, (3) 15 μM Cu, and (4) 30 μM Ni and 15 μM Cu. Each point represents the average of 3 replicates and error bars represent ±SD.


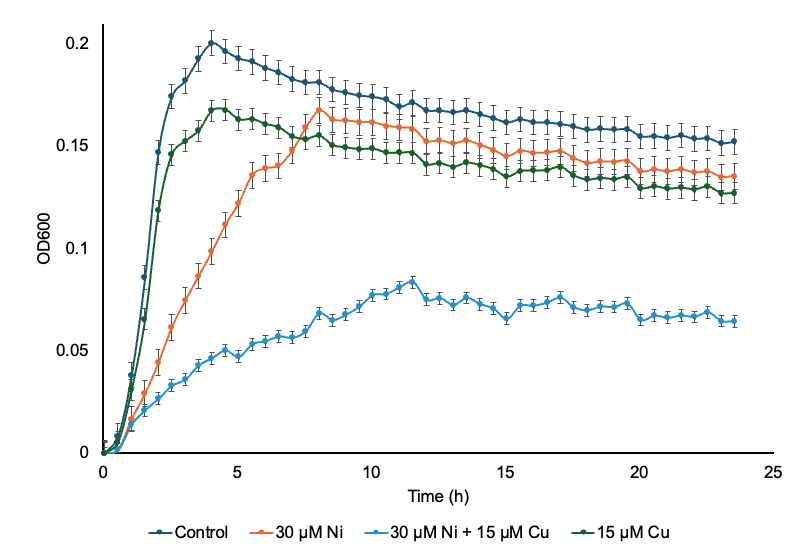


**Figure S2.** Growth curves of the wild-type strain of E. coli under the following conditions: (1) a control (no treatment), (2) 30 μM Ni, (3) 15 μM Cu, and (4) 30 μM Ni and 15 μM Cu. Each point represents the average of 3 replicates and error bars represent ±SD. This figure shows an example of the increased lag time that was sometimes observed in the Ni-exposed cultures.


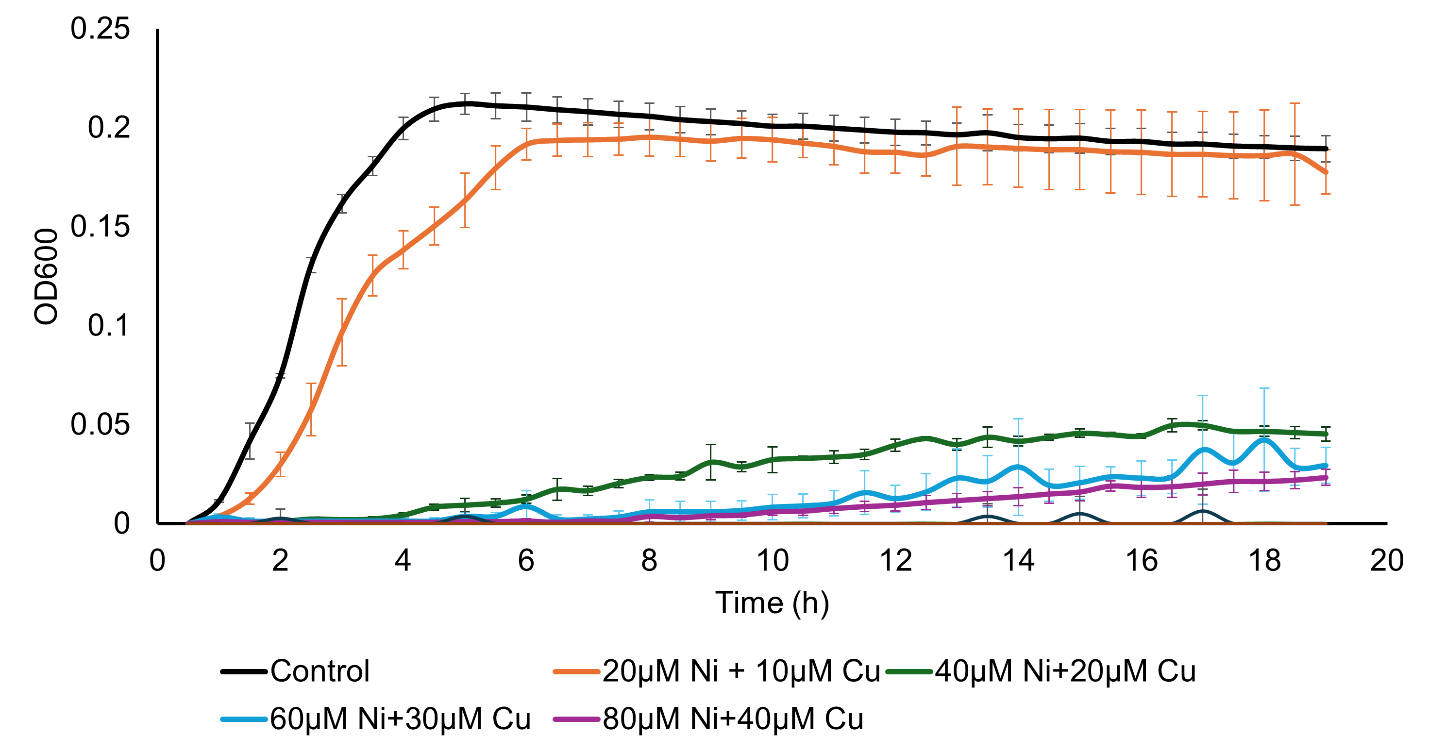


**Figure S3.** Growth curves of E. coli under control conditions and exposure to different environmentally relevant combinations of Ni and Cu. Each point represents the average of 3 replicates and error bars represent ±SD.

***
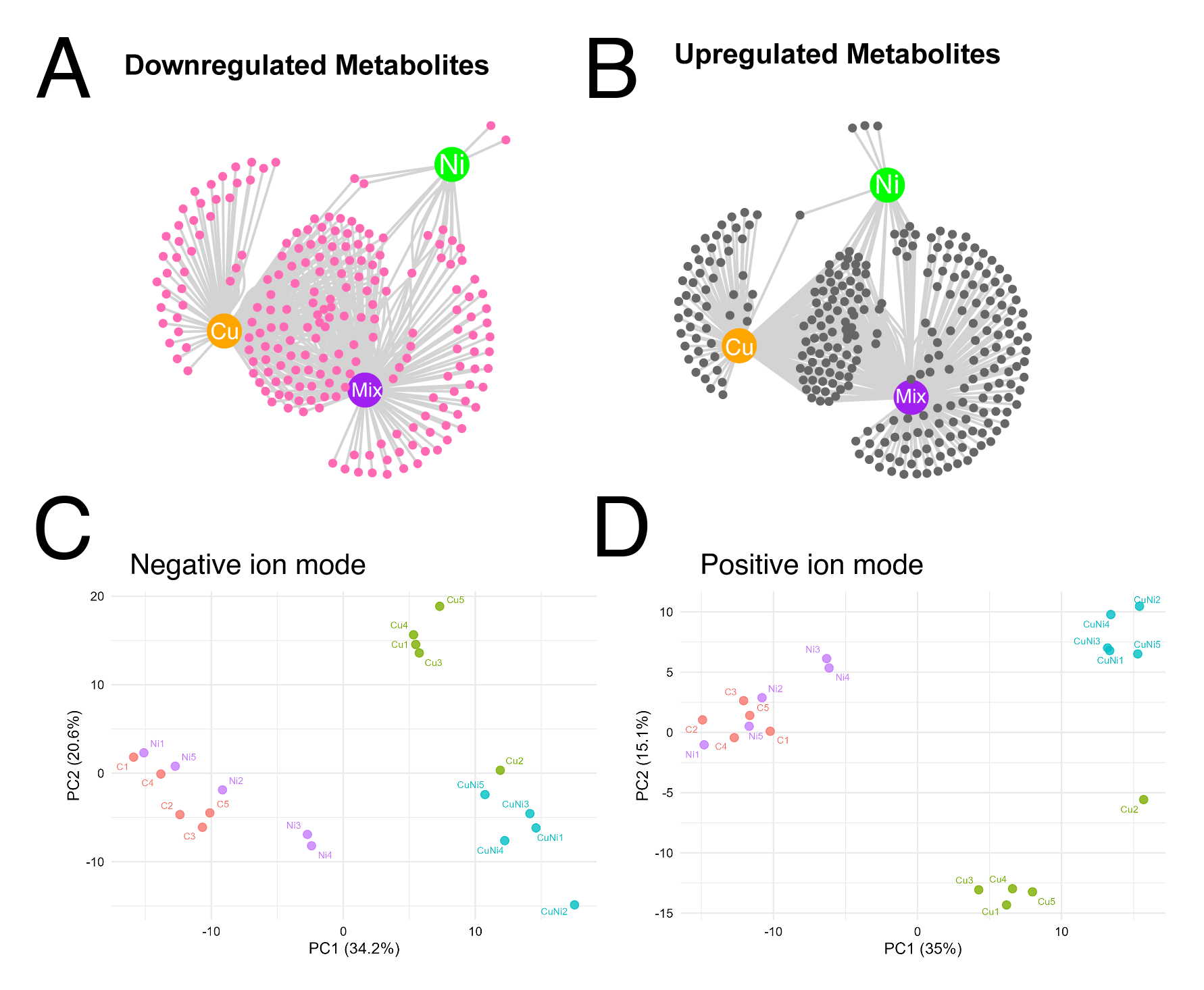
***

**Figure S4.** Metabolomic analysis of E. coli reveals metabolic changes induced by 15 µM Cu, 30 µM Ni, and their combination. (A) Network diagram of downregulated metabolites. (B) Network diagram of upregulated metabolites. The network diagram displays differentially abundant metabolites (small nodes) in response to each metal treatment (large, colored nodes). Pink small nodes represent downregulated metabolites, while gray small nodes represent upregulated metabolites. (C) PCA plot of negative ion mode metabolite counts. (D) PCA plot of positive ion mode metabolite counts. Metabolomics data can be found in **Table S4**.

**Table S5. Changes in expression of the OxyR regulon**

| **gene** | **Combined Cu and Ni (Log2FC)** | **Cu (Log2FC)** | **Ni (Log2FC)** |
| --- | --- | --- | --- |
| *hemH* | No change | No change | No change |
| *ahpC* | No change | No change | No change |
| *ahpF* | No change | No change | No change |
| *dsbG* | No change | No change | No change |
| *fur* | No change | No change | No change |
| *dps* | No change | No change | No change |
| *grxA* | No change | No change | No change |
| *ybjC* | No change | No change | No change |
| *nfsA* | No change | No change | No change |
| *rimK* | No change | No change | No change |
| *ybjN* | No change | No change | No change |
| *hcp* | -1.58 | No change | +1.57 |
| *hcr* | No change | No change | No change |
| *poxB* | No change | No change | No change |
| *ychF* | No change | No change | No change |
| *sufA* | No change | No change | No change |
| *sufB* | No change | No change | No change |
| *sufC* | No change | No change | No change |
| *sufD* | No change | No change | No change |
| *sufS* | No change | No change | No change |
| *sufE* | No change | No change | No change |
| *znuC* | No change | No change | No change |
| *znuB* | No change | No change | No change |
| *znuA* | -2.05 | No change | No change |
| *zinT* | No change | No change | No change |
| *elaB* | No change | No change | No change |
| *mntH* | +3.11 | No change | -1.68 |
| *hemF* | No change | No change | No change |
| *trxC* | +1.61 | No change | No change |
| *gor* | No change | No change | No change |
| *ccp* | No change | No change | No change |
| *metR* | +3.36 | No change | No change |
| *metE* | +2.78 | No change | No change |
| *katG* | No change | No change | No change |
| *oxyS* | -1.61 | No change | No change |
| *oxyR* | No change | No change | No change |
| *gntP* | No change | No change | No change |


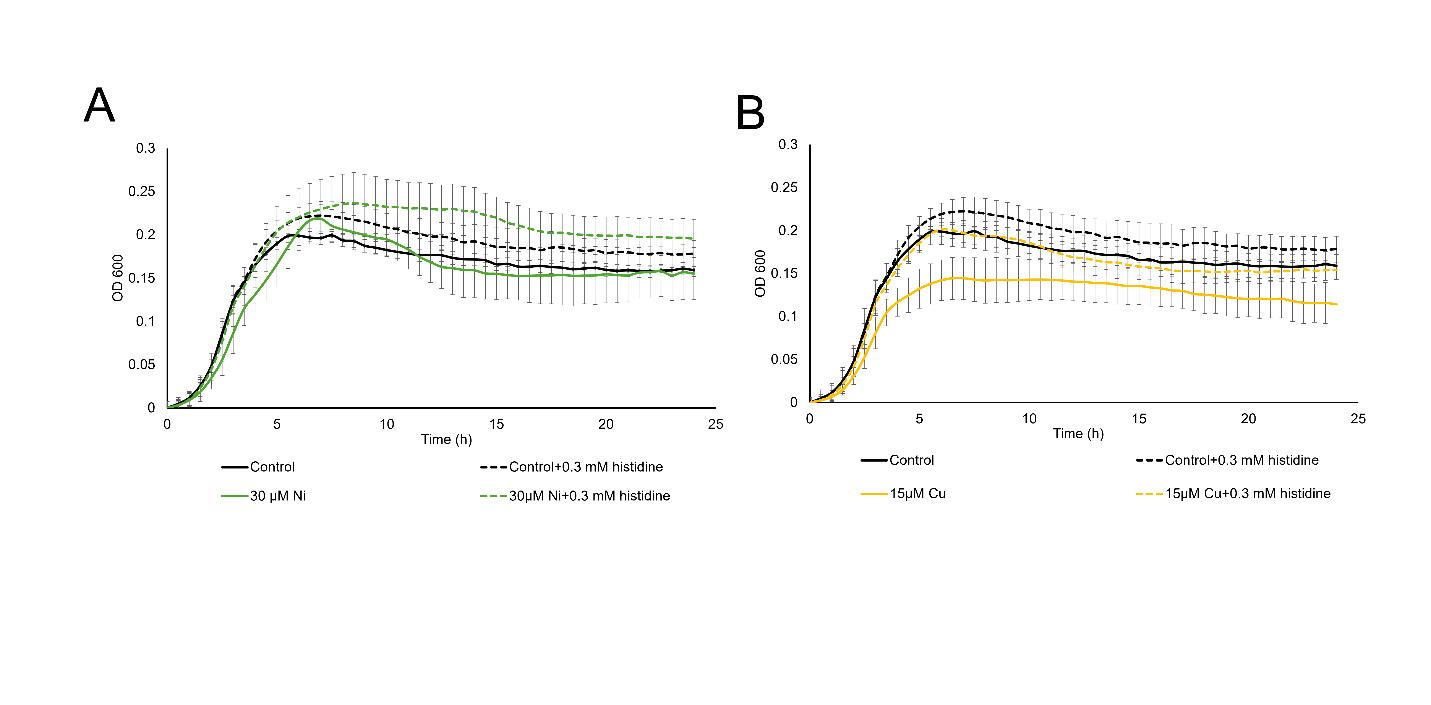


**Figure S5.** Effect of histidine supplementation on E. coli growth under single metal exposure (Ni or Cu stress). Each point represents the average of 3 replicates and error bars represent ±SD. (A) Growth under 30 µM Ni with and without 0.3 mM histidine, compared to control (± histidine). (B) Growth under 15 µM Cu with and without 0.3 mM histidine, compared to control (± histidine).

**
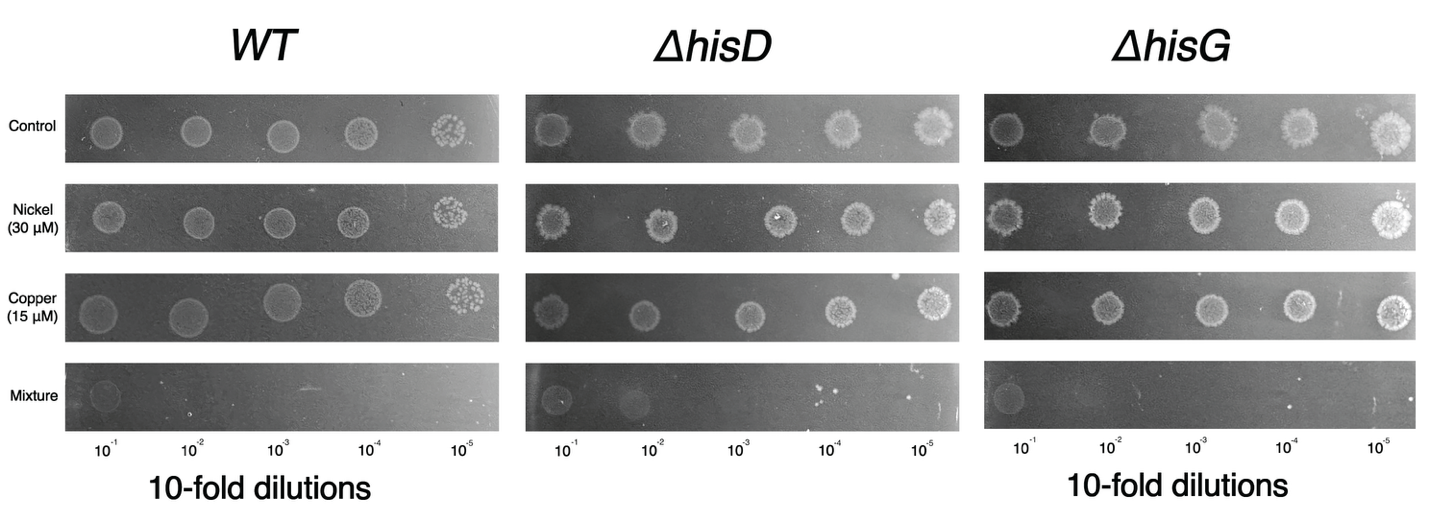

Figure S6.** Spot dilution assay reveals metal sensitivity of histidine biosynthesis mutants in E. coli. Serial 10-fold dilutions (10⁻¹ to 10⁻⁵) of wild-type *E. coli, ΔhisD* *ΔhisG* mutant strains were spotted onto MES minimal agar plates under four conditions: untreated control, 30 µM Ni, 15 µM Cu and a combined treatment of both metals. Experiments were performed a minimum of two times, with one representative trial shown.


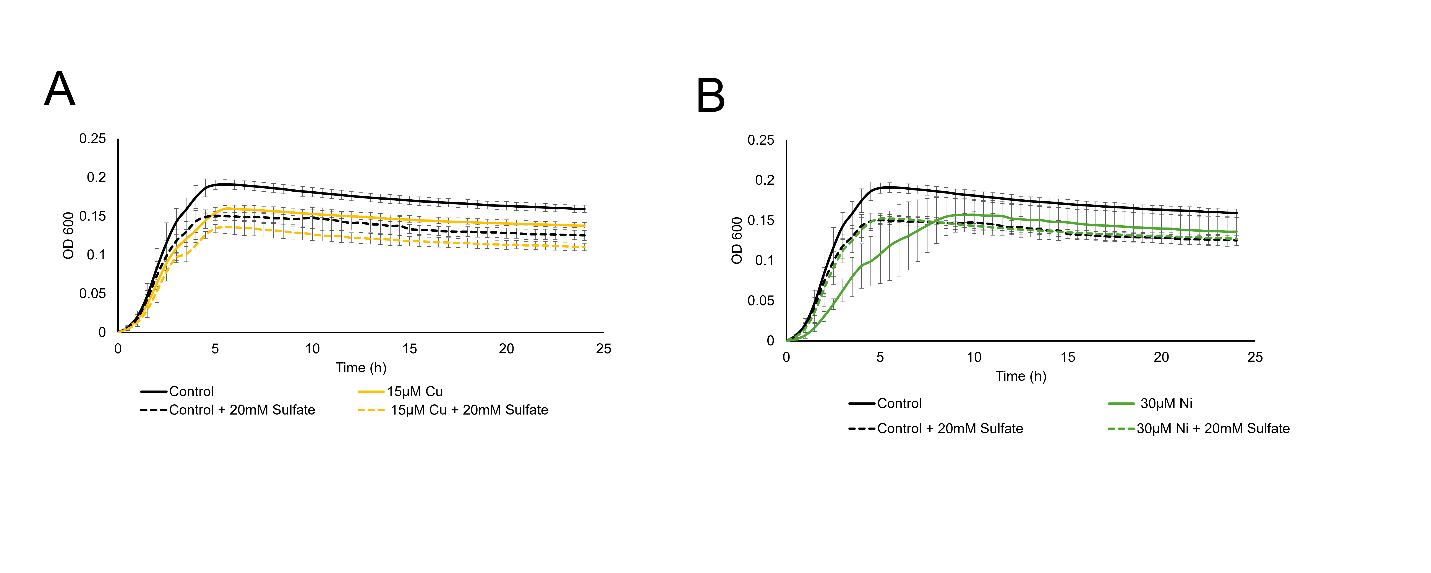


**Figure S7.** Effect of sulfate supplementation on E. coli growth under single metal exposure (Ni or Cu stress). Each point represents the average of 3 replicates and error bars represent ±SD. (A) Growth under 30 µM Ni with and without 20 mM sulfate, compared to control (± sulfate). (B) Growth under 15 µM Cu with and without 20 mM sulfate, compared to control (± sulfate).


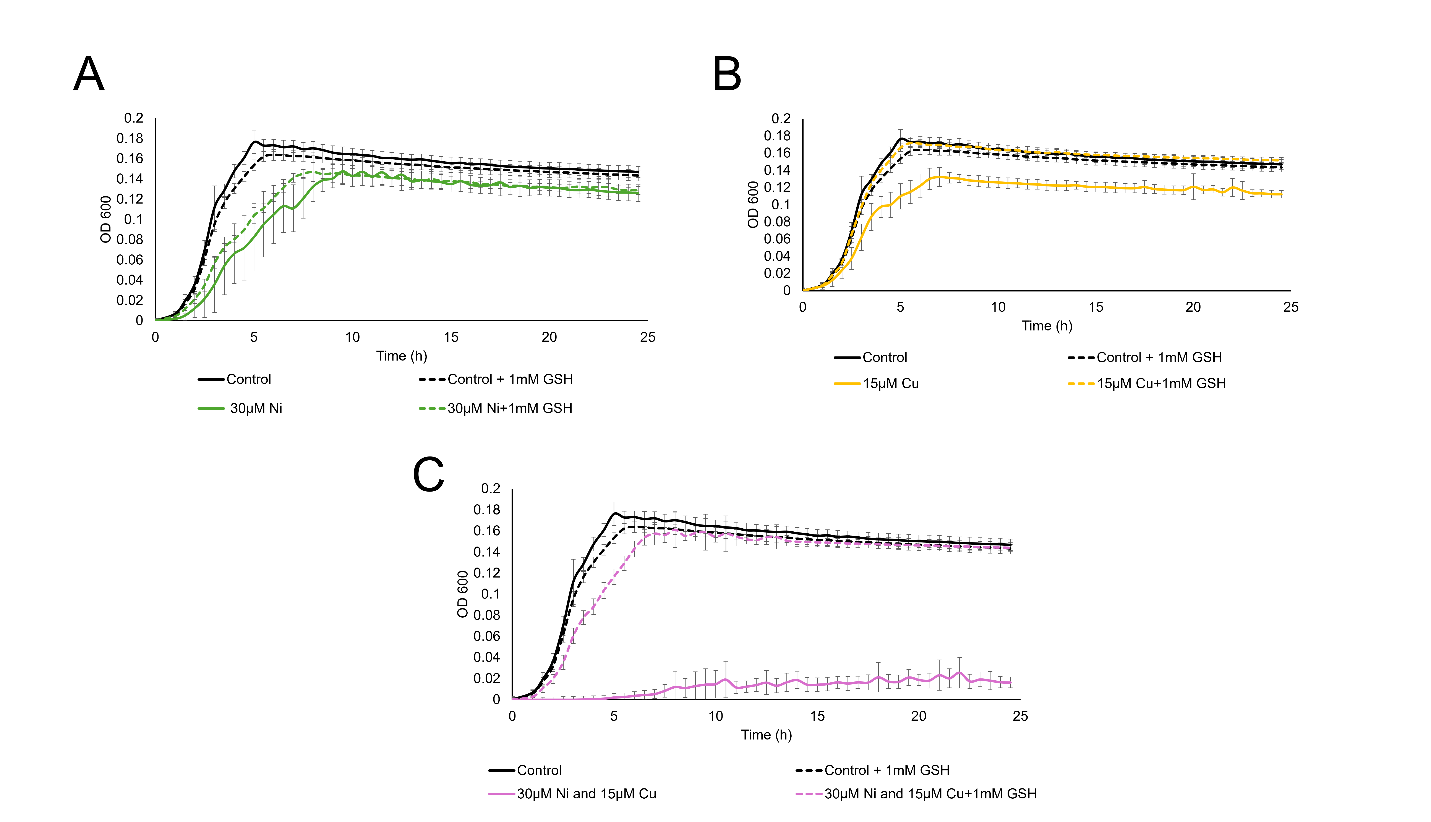


**Figure S8**. Effect of glutathione supplementation on E. coli growth metal exposure (Ni and/or Cu stress). Each point represents the average of 3 replicates and error bars represent ±SD. (A) Growth under 30 µM Ni with and without 1mM GSH, compared to control (± GSH). (B) Growth under 15 µM Cu with and without 1mM GSH, compared to control (± GSH). (C) Growth under 30 µM Ni and 15 µM Cu with and without 1mM GSH, compared to control (± GSH).


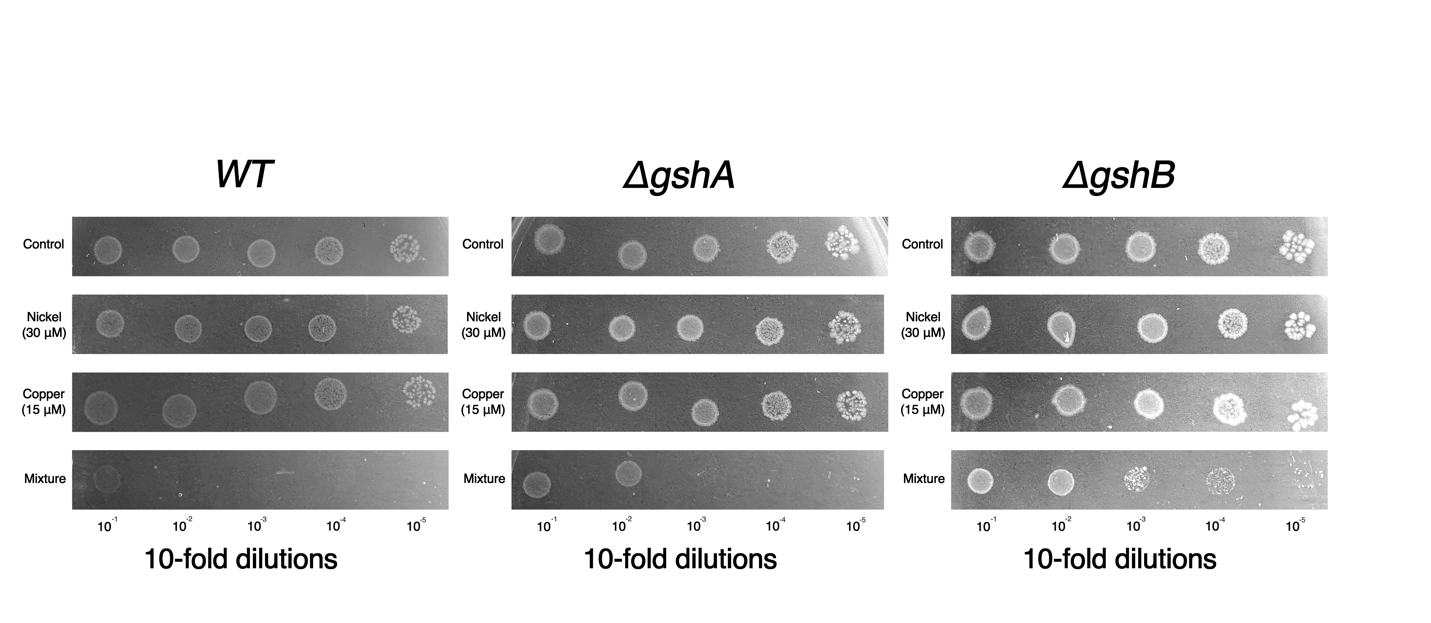


**Figure S9**. Spot dilution assay reveals metal sensitivity of glutathione biosynthesis mutants in E. coli. Serial 10-fold dilutions (10⁻¹ to 10⁻⁵) of wild-type E. coli, ΔgshA ΔgshB mutant strains were spotted onto MES minimal agar plates under four conditions: untreated control, 30 µM Ni, 15 µM Cu and a combined treatment of both metals. Experiments were performed a minimum of two times, with one representative trial shown.


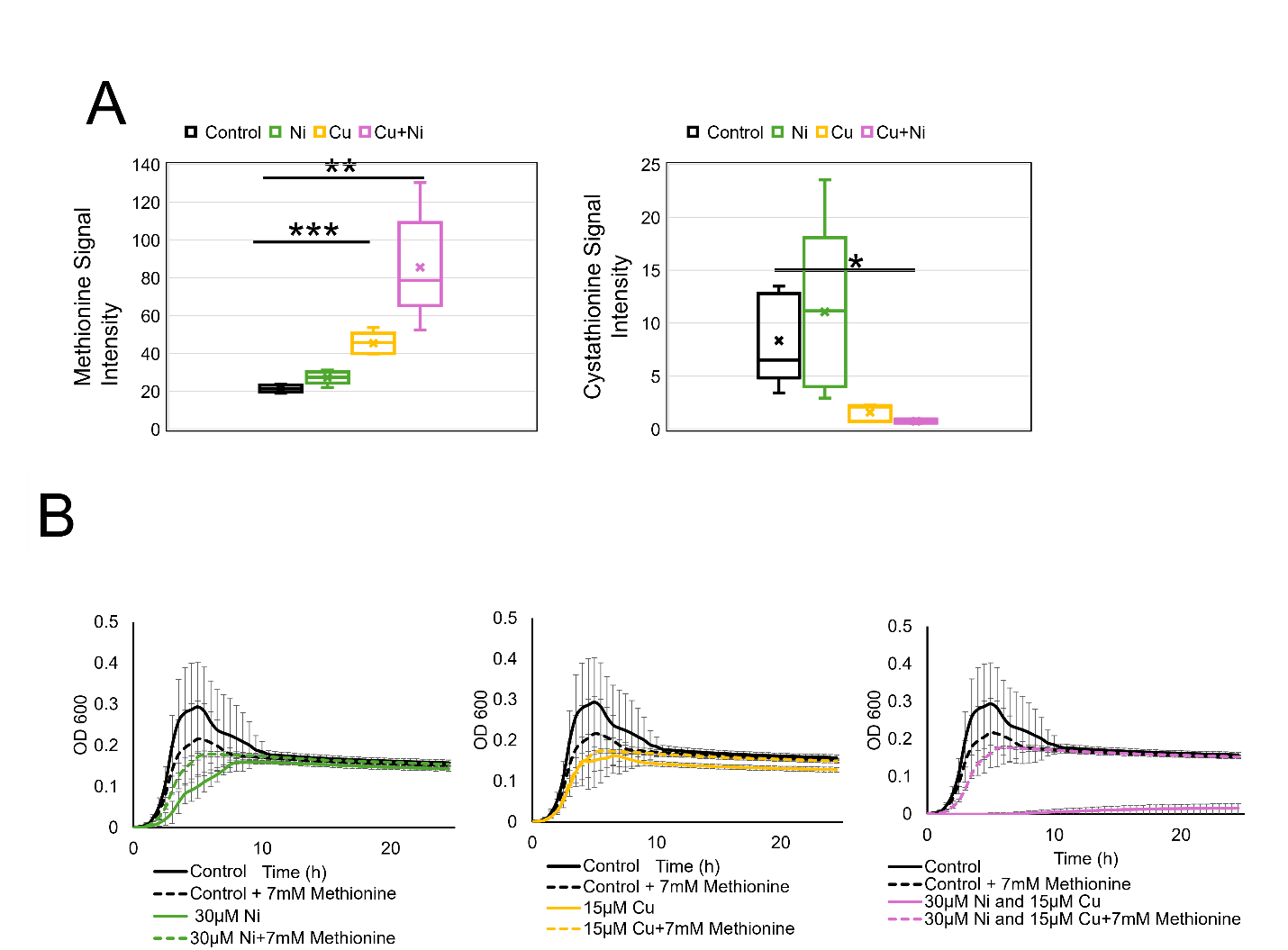


**Figure S10**. Methionine levels are altered by metal stress and supplementation improves E. coli growth under metal stress. (A) Relative methionine and cystathionine signal intensity measured by metabolomic analysis in E. coli cultures treated with 30 µM Ni, 15 µM Cu, and their combination (Ni+Cu), compared to the untreated control. Statistical significance: p<0.05 (*); p < 0.01 (**); p < 0.001 (***). (B) Growth curves of E. coli under each treatment condition (Ni, Cu, Ni+Cu) compared to control, with or without supplementation of 7 mM methionine. Each point represents the average of 3 replicates and error bars represent ±SD


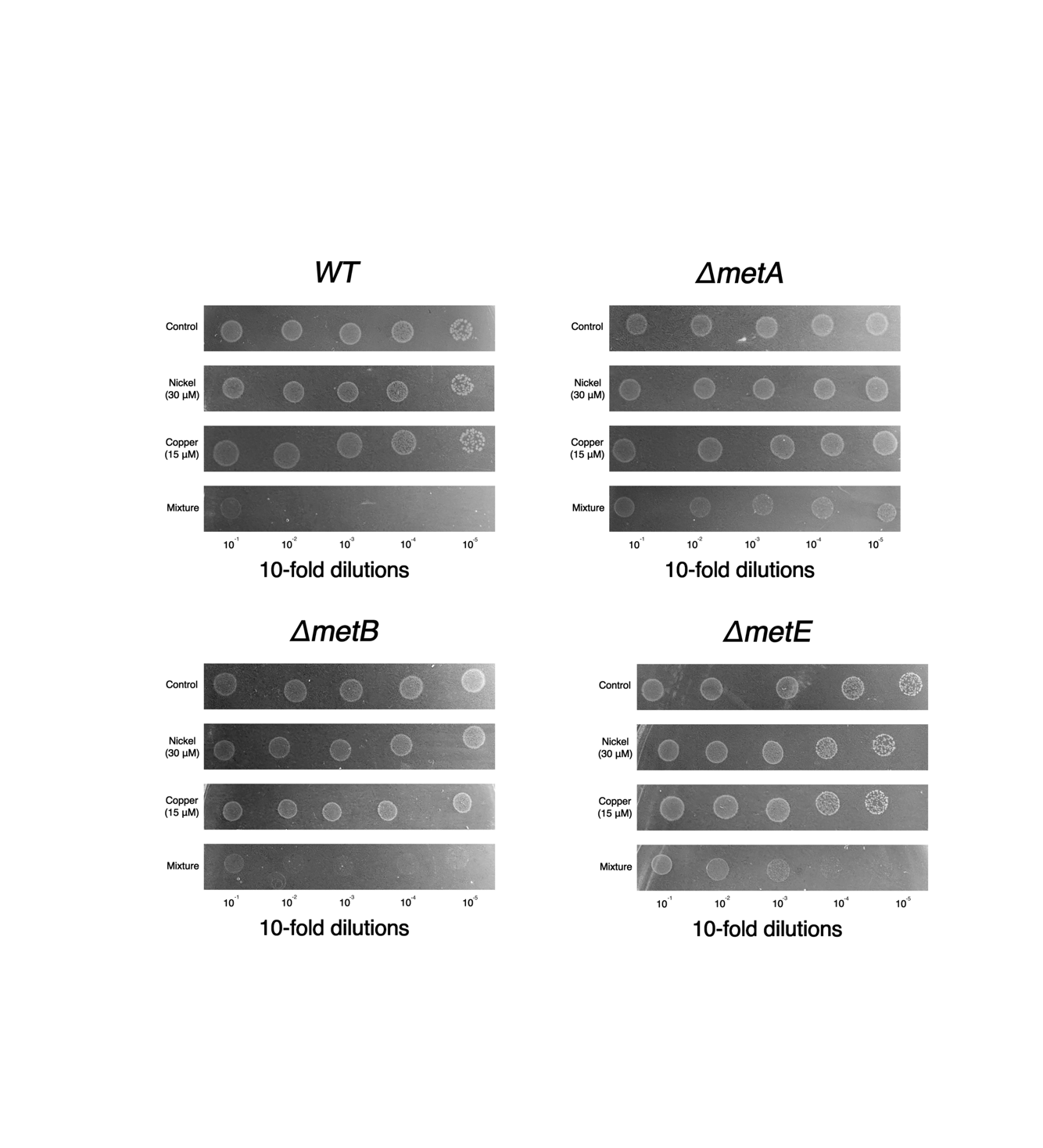


**Figure S11**. Growth of methionine biosynthesis mutants under metal stress conditions. Spot dilution assay comparing wild-type *E. coli* and *ΔmetA, ΔmetB*, and *ΔmetE* mutants on MES minimal agar. Serial 10-fold dilutions (10⁻¹ to 10⁻⁵) of wild-type *E. coli, ΔmetA*, *ΔmetB*, and *ΔmetE* mutant strains were spotted onto MES minimal agar plates under four conditions: untreated control, 30 µM Ni, 15 µM Cu, and a combined treatment of both metals. Experiments were performed a minimum of two times, with one representative trial shown. Note that a single wild-type strain experiment is shown across figure panels, for ease of comparison, since experiments were performed in parallel.


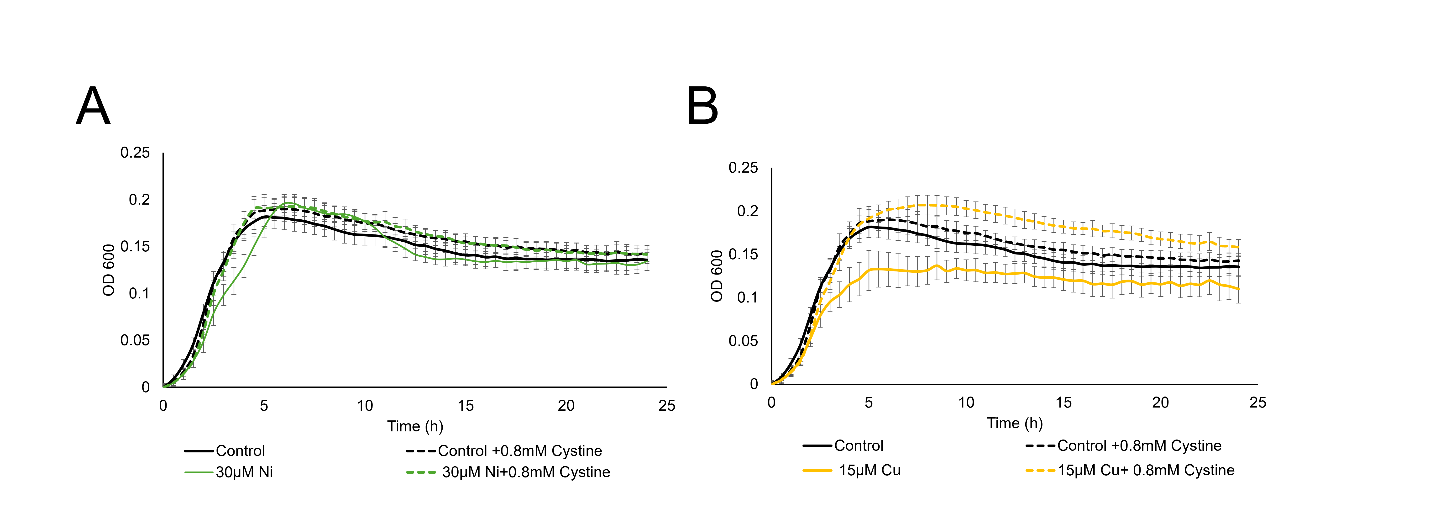


**Figure S12.** Effect of cystine supplementation on E. coli growth under single metal exposure (Ni or Cu stress). Each point represents the average of 3 replicates and error bars represent ±SD. (A) Growth under 30 µM Ni with and without 0.8 mM cystine, compared to control (± cystine). (B) Growth under 15 µM Cu with and without 0.8 mM cystine, compared to control (± cystine).


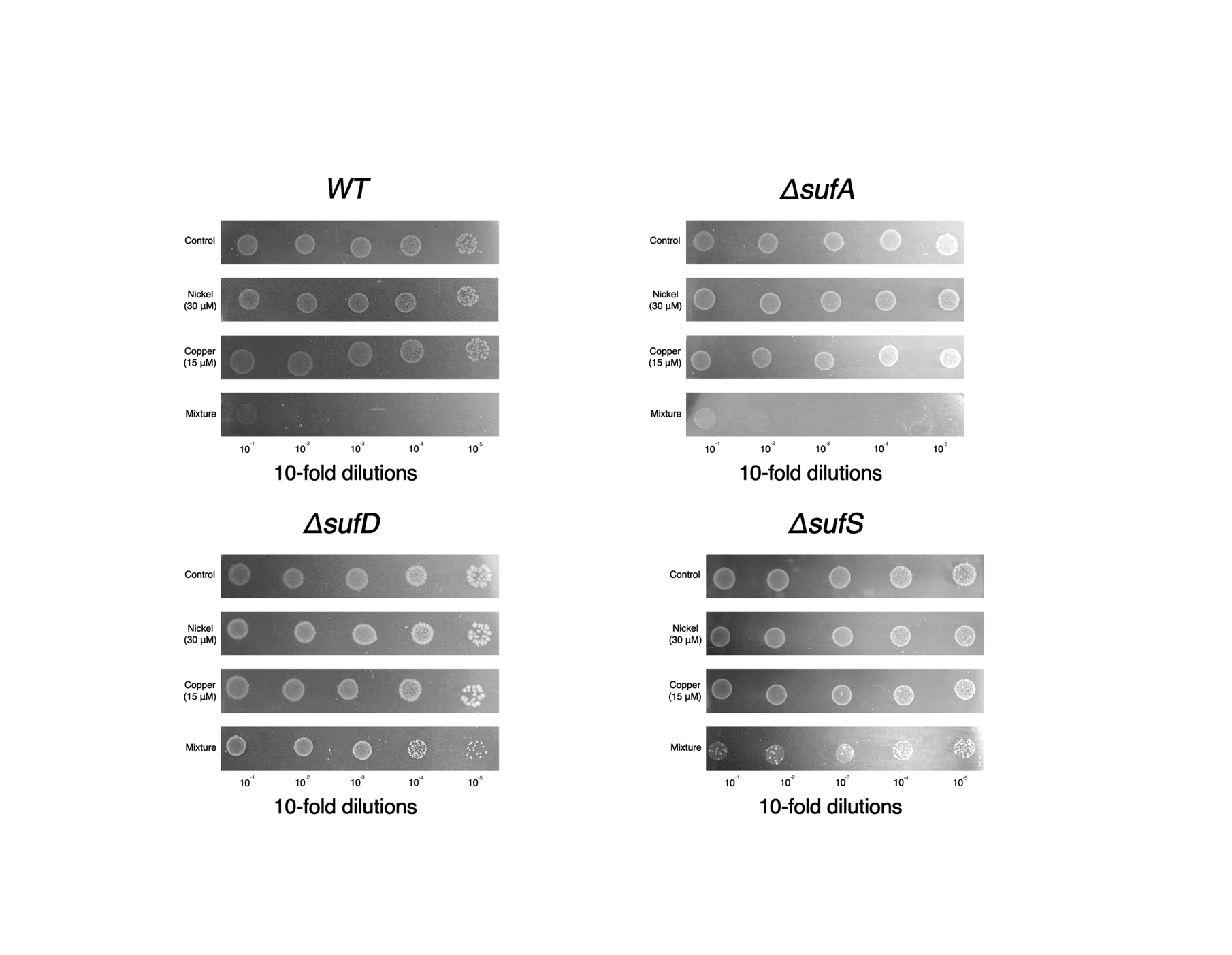


**Figure S13.** Growth of SUF pathway mutants under metal stress conditions. Spot dilution assay comparing wild-type *E. coli* and *∆sufA*, *∆sufD*, and *∆sufS* mutants on MES minimal agar. Serial 10-fold dilutions (10⁻¹ to 10⁻⁵) of wild-type *E. coli*, *∆sufA, ∆sufD,* and *∆sufS* strains were spotted onto MES minimal agar plates under four conditions: unsupplemented control, 30 µM Ni, 15 µM Cu, and a combined treatment of both metals. Experiments were performed a minimum of two times, with one representative trial shown. Note that a single wild-type strain experiment is shown across figure panels, for ease of comparison, since experiments were performed in parallel.

**
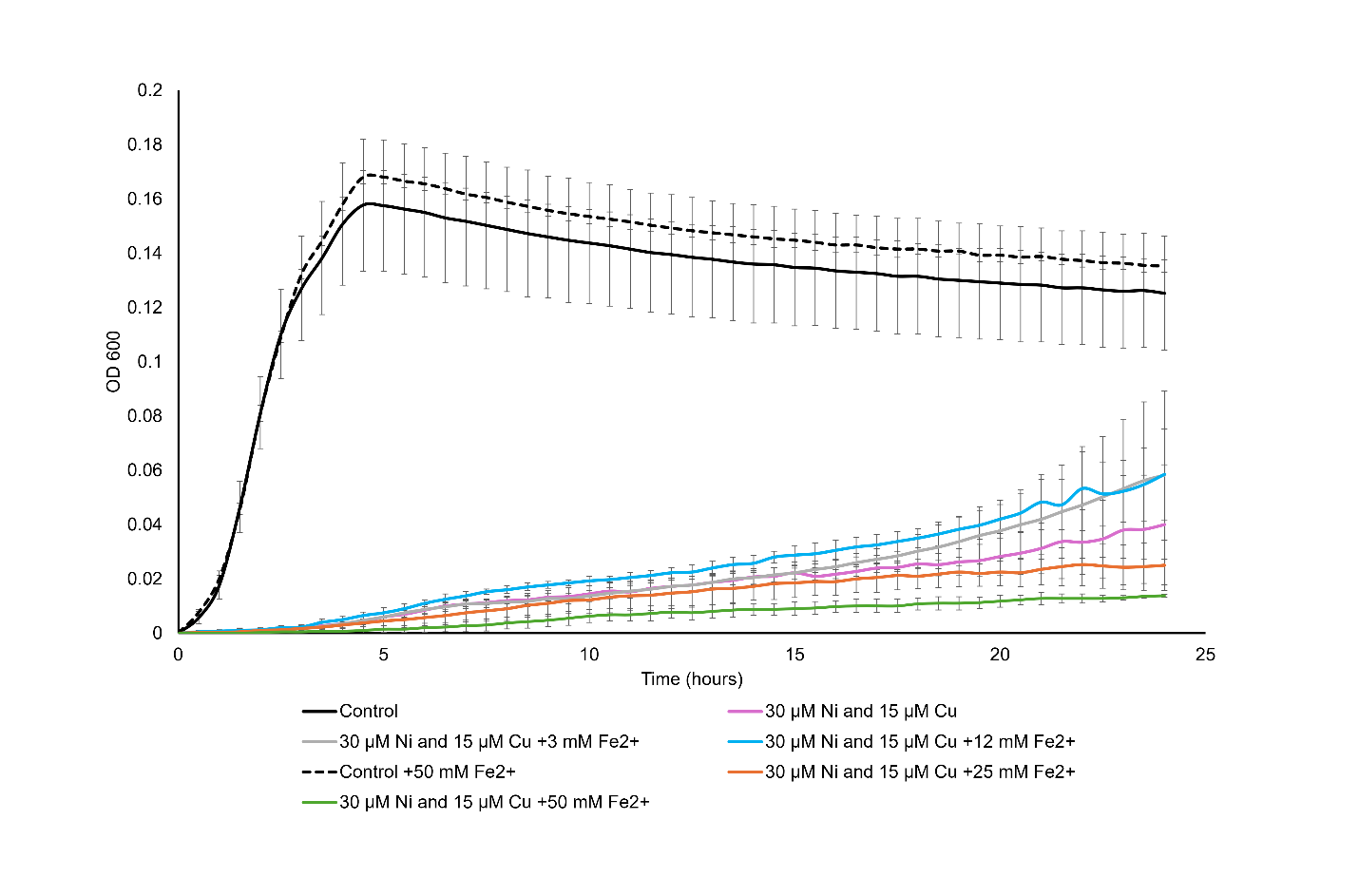
**

**Figure S14.** Effect of ferrous iron supplementation (Fe^2+^) supplementation on E. coli under 30 µM Ni and 15 µM Cu exposure and compared to the control (± Fe^2+^). Each point represents the average of 3 replicates and error bars represent ±SD. Ferrous iron was chelated with citrate in a 3:2 molar ratio to prevent oxidation to Fe^3+^.


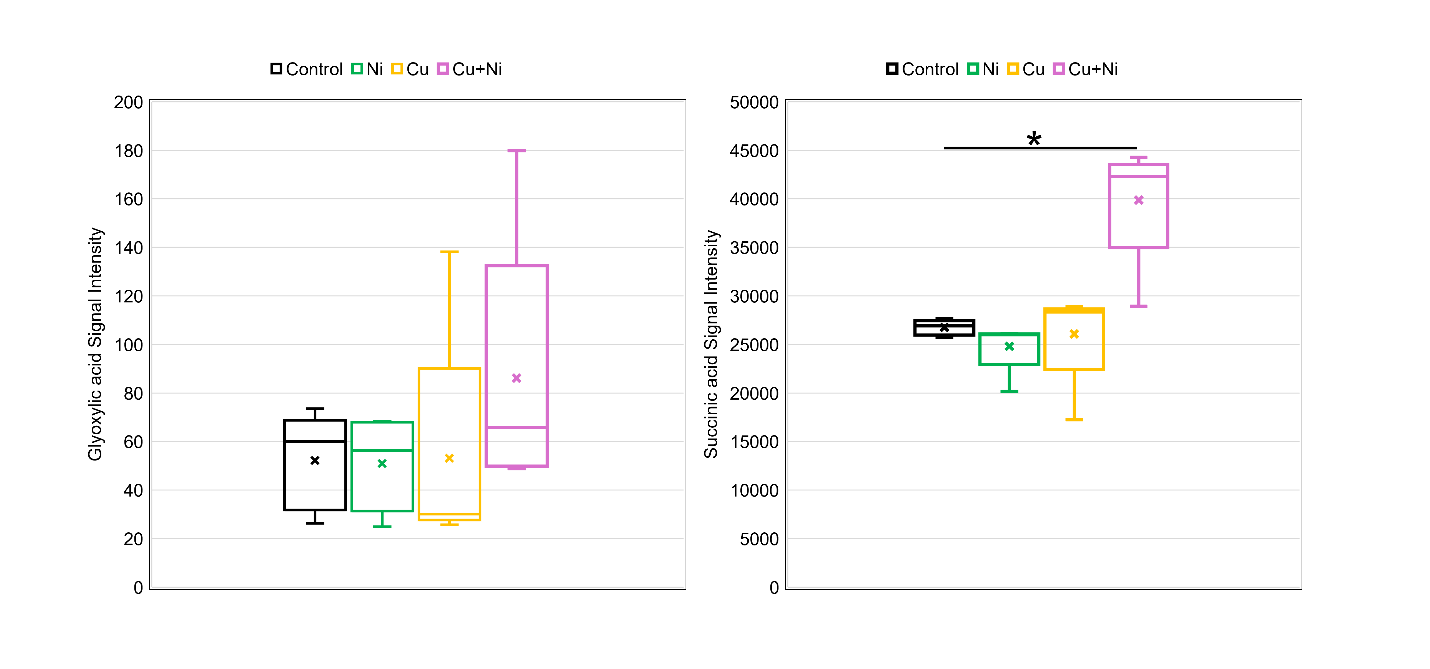


**Figure S15.** Relative glyoxylic acid and succinic acid signal intensity measured by metabolomic analysis in *E. coli* cultures treated with 30 µM Ni, 15 µM Cu, and their combination (Ni+Cu), compared to the untreated control. Statistical significance: p < 0.05 (*).


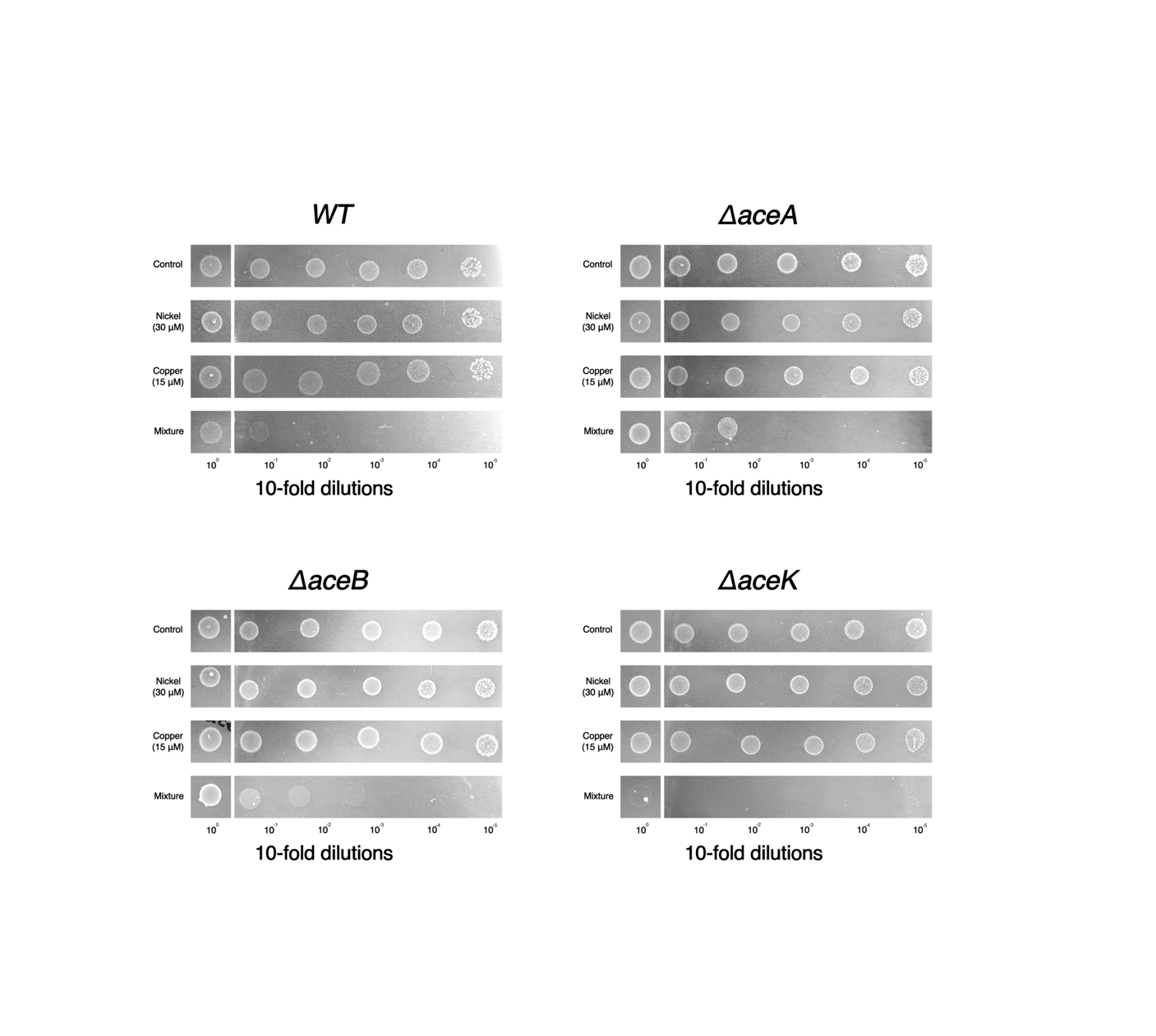


**Figure S16. Growth of glyoxylate shunt mutants under metal stress conditions.**Spot dilution assay comparing wild-type E. coli and *∆****aceA***, *∆****aceB***, and *∆****aceK*** mutants on MES minimal agar. Serial 10-fold dilutions (10⁻¹ to 10⁻⁵) of wild-type E. coli, *∆****aceA***, *∆****aceB***, and *∆****aceK*** strains were spotted onto MES minimal agar plates under four conditions: unsupplemented control, 30 µM Ni, 15 µM Cu, and a combined treatment of both metals. Experiments were performed at least twice, with one representative trial shown. Note that a single wild-type strain experiment is shown across figure panels, for ease of comparison, since experiments were performed in parallel.
